# A universal foundation model for transfer learning in molecular crystals†

**DOI:** 10.1039/d5sc00677e

**Published:** 2025-05-21

**Authors:** Minggao Feng, Chengxi Zhao, Graeme M. Day, Xenophon Evangelopoulos, Andrew I. Cooper

**Affiliations:** a Materials Innovation Factory and Department of Chemistry, University of Liverpool Liverpool UK evangx@liverpool.ac.uk aicooper@liverpool.ac.uk; b School of Chemistry and Chemical Engineering, University of Southampton Southampton UK g.m.day@soton.ac.uk; c Leverhulme Research Centre for Functional Materials Design Liverpool UK

## Abstract

The physical and chemical properties of molecular crystals are a combined function of molecular structure and the molecular crystal packing. Specific crystal packings can enable applications such as pharmaceuticals, organic electronics, and porous materials for gas storage. However, to design such materials, we need to predict both crystal structure and the resulting physical properties, and this is expensive using traditional computational methods. Machine-learned interatomic potential methods offer major accelerations here, but molecular crystal structure prediction remains challenging due to the weak intermolecular interactions that dictate crystal packing. Moreover, machine-learned interatomic potentials do not accelerate the prediction of all physical properties for molecular crystals. Here we present Molecular Crystal Representation from Transformers (MCRT), a transformer-based model for molecular crystal property prediction that is pre-trained on 706 126 experimental crystal structures extracted from the Cambridge Structural Database (CSD). MCRT employs four different pre-training tasks to extract both local and global representations from the crystals using multi-modal features to encode crystal structure and geometry. MCRT has the potential to serve as a universal foundation model for predicting a range of properties for molecular crystals, achieving state-of-the-art results even when fine-tuned on small-scale datasets. We demonstrate MCRT's practical utility in both crystal property prediction and crystal structure prediction. We also show that model predictions can be interpreted by using attention scores.

## Abstract 2

Multi-modal transfer learning for predicting and explaining universal properties of organic crystals.

## Graphical abstract

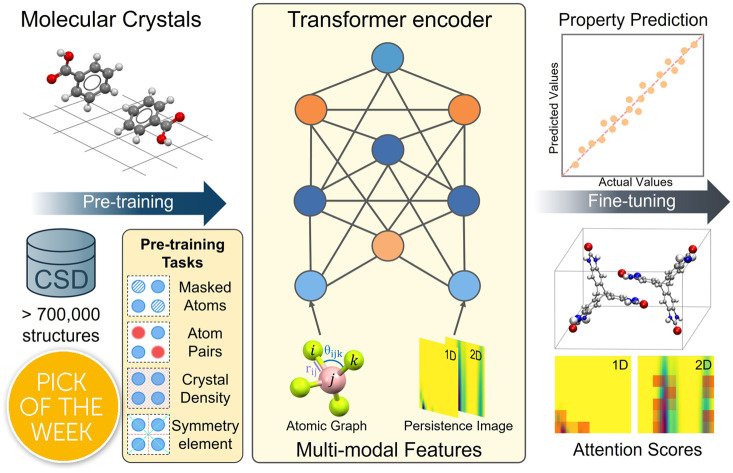

## 1 Introduction

Molecular crystals have diverse applications including pharmaceuticals,^1^ organic electronics,^2^ optical materials,^3^ and materials for gas storage and separation.^4–6^ In all cases, the properties of molecular crystals depend on the crystal packing. For example, pharmaceutical molecules can have widely different solubilities depending on the crystalline form, and in organic electronics, charge transport is critically dependent on crystal packing. However, molecular crystal packing is notoriously difficult to predict because it is dictated by a range of weak intermolecular interactions, such as van der Waals forces, aromatic pi-stacking, and hydrogen bonds.^7^ This is a major hurdle for digital material design because if we cannot predict crystal structure then we cannot, by definition, predict the functional properties of the crystal. To address this challenge, crystal structure prediction (CSP) methods have been created to identify molecular crystals with specific target functionalities. For example, energy–structure–function (ESF) maps have guided the synthesis of various functional molecular crystals.^6–10^ However, despite these successes, calculating the physical properties for each structure on an ESF map, or even a sub-set of low-energy structures, can be computationally demanding. This problem is two-fold: the prediction of lattice energy, or crystal stability, is itself computationally expensive, and the functional property calculations are usually even more expensive. To tackle this, there has been a surge of interest in machine learning (ML) techniques for the rapid prediction of materials properties and the elucidation of structure-property relationships^11,12^ at a fraction of the cost of first-principles methods, such as density functional theory (DFT).

Learning accurate representations is a crucial aspect of machine learning theory that also extends to learning molecular representations. Different types of materials pose different challenges when learning accurate latent representations. For example, in solid-state systems it is essential to capture features such as long-range interactions and periodicity in property prediction tasks.^13^ This is especially challenging in organic molecular crystals due to the intermolecular interactions,^7^ which are typically weaker than for ionic inorganic materials. Hence, many of the inherently local graph-based deep architectures fail to capture global-driven properties, while traditional ML models that use hand-crafted descriptors have been more successful in capturing spatial information in some cases.^14,15^ Hand-crafted descriptors such as smooth overlap of atomic positions (SOAP)^16^ and atom-centered symmetry functions (ACSFs)^17^ have proven effective in predicting properties like lattice energy,^12^ while geometric descriptors such as accessible surface area and pore diameters have been used to predict the methane deliverable capacity of molecular crystals^18^ as well as other global-driven properties of porous materials.^19^ More recently, persistent homology^20,21^ was shown to encode global molecular geometric features into machine-learned representations.^22^ Nevertheless, calculating descriptors such as SOAP can be cumbersome in terms of memory footprint for larger organic systems, whereas geometric descriptors tend to overly compress the geometric information of the crystals, failing to adequately encode the fine detail of the molecular geometry. Another fundamental drawback of deep learning models is the need for re-training and hyperparameter re-optimization for each specific problem and property, adding further time and computational cost. A further challenge is the availability of training data: ideally, we need methods that can be fine-tuned on small scale datasets, because for chemistry problems, data is often scarce and expensive.

Transfer learning allows a model trained on one task to be adapted to a different task, significantly reducing the need for extensive retraining. Universality is a key aspect of a pre-trained model to allow it to capture simultaneously molecular features of varying modalities, as well as local and global interactions. Recently, pre-trained models using transformers^23^ have been designed for metal–organic frameworks (MOFs) and showed exceptional performance across a range of different tasks.^24–27^ Transformers enable multi-modal input integration combined with self-attention layers that can process data sequences in parallel, allowing for much more efficient training routines. Also, the attention scores (AS) within the self-attention layers can be used to analyse feature importance and thus offer an interpretability tool to gain insights on the prediction process itself, unlike other black-box learning systems. A leading example is BERT,^28^ a pre-trained language transformer model that shows state-of-the-art results across various downstream tasks after being trained on large-scale data. More recently, vision transformers architectures (ViTs)^29^ have paved the way for the integration of multi-modal inputs towards more universal models^30–32^ and inspired a number of recent works in materials science.^24–27,33–35^

There are two key challenges when designing the pre-training framework of a universal transformer model, namely the choice of multi-modal input features and the design of pre-training tasks. The choice of appropriate input features is crucial to enrich the representation capacity of the pre-trained model so it is applicable across a wide-range of tasks, while the pre-training tasks should be designed carefully to efficiently but accurately capture both local and global interactions across the training set. The design of a pre-training framework is challenging for organic molecular crystals that are defined by a range of inter- and intra-molecular interactions of widely varying strength and directionality, combined with geometric information about symmetry and molecular packing.^36^

Here we introduce a foundation model focused on molecular crystal structures that can be used as a universal tool for a wide range of prediction tasks for materials applications that would otherwise require time-consuming calculations. We present a novel transformer-based pre-training framework—Molecular Crystal Representation from Transformers (MCRT)—that has been pre-trained on a dataset of 706 126 experimentally-determined structures sourced from the Cambridge Structural Database (CSD).^37^ MCRT accommodates multi-modal inputs that encode both local and global features in conjunction with a set of carefully designed pre-training tasks that help capture universal representations for predicting a wide range of different crystal properties, achieving state-of-the-art performance. We tested MCRT's performance on a range of prediction tasks on crystalline properties such as lattice energy, methane deliverable capacity (as relevant for natural gas-powered vehicles), diffusivity, bulk modulus (relevant to the tabletting of pharmaceuticals) and charge mobility (relevant in organic electronics), thus demonstrating that the model can be applied to both porous and non-porous organic solids. We further explored different ablations of the proposed model, as well as its learning capacity limits under data scarcity conditions. Importantly, MCRT's attention-based architecture allows us to gain a more intuitive understanding of the structure–property relationships in molecular crystals through cumulative attention scores^38^ from across the different layers of MCRT.

## 2 Results and discussion

### 2.1 Overview of pre-training framework

**Fig. 1:**
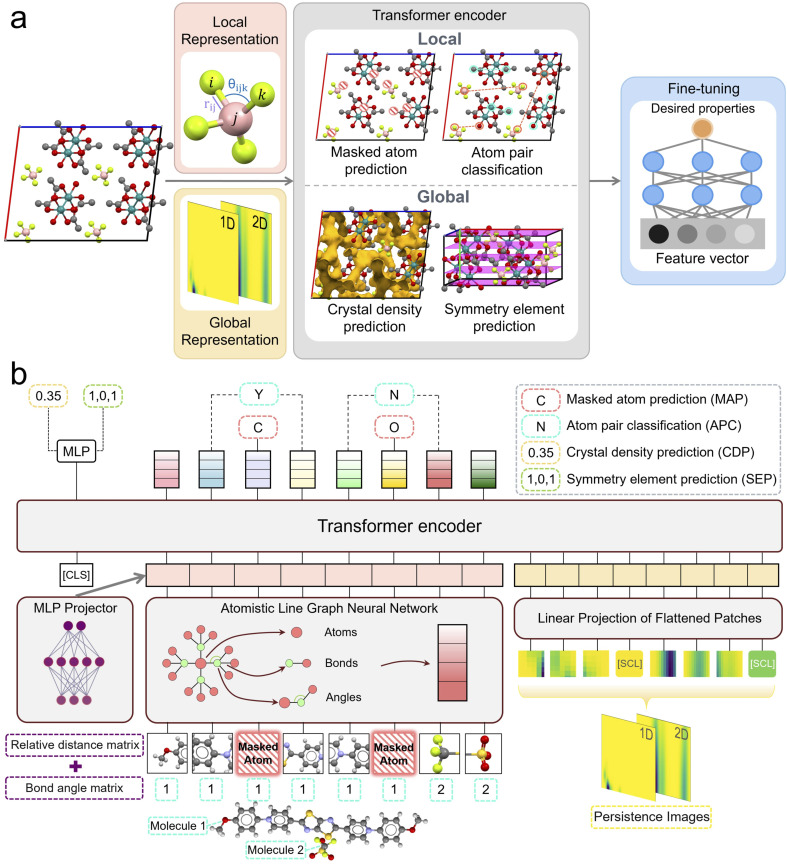
Schematic overview of MCRT framework. (a) Molecular crystals are represented using local and global features, which are then fed into the model. During the pre-training phase, the model undergoes pre-training on four tasks: masked atom prediction (MAP), atom pair classification (APC), crystal density prediction (CDP), and symmetry element prediction (SEP). In the fine-tuning phase, the model is initialised with parameters from the pre-trained model and a simple prediction head is added to train for the desired properties of molecular crystals. (b) Architecture for pre-training MCRT. Before being fed into the model, 15% of the atoms are randomly masked, and the model is tasked with predicting the types of the masked atoms based on the final atomic representations. Each atom is pre-assigned a molecular label, indicating which molecule within the *P*1 unit cell it belongs to, thus providing labels for atom pairs in the subsequent APC task. Meanwhile, SEP and CDP tasks, as global pre-training tasks, leverage the output of the [CLS] token representing the entire crystal for their predictions.

The overall framework of MCRT is illustrated in Fig. 1a. It comprises a transformer encoder module that is used to build a pre-trained model that then acts as foundation for fine-tuning on a range of downstream prediction tasks. The pre-trained model was built with universality in mind, and designed to distill critical features of molecular crystals without the need for labeled data and, subsequently, to extrapolate desirable physical properties across various applications after fine-tuning. This transformer is therefore designed as a multi-modal architecture that processes two distinct input modalities that encode both local and global information: atom-based graph embeddings and persistence image embeddings.

#### 2.1.1 Atom-based graph embeddings

These are embeddings taken from the penultimate layer of an ALIGNN architecture,^39^ which performs message passing on both the interatomic bond graph and its line graph corresponding to bond angles, thus integrating bond length and angle information to provide a more enriched representation of the local environments in a crystal structure. To further enhance the positional information of each atom and to support an efficient training process, we added relative positional embeddings to the atomic features (Fig. S2†), which were integrated with the atomic features before being fed into the transformer encoder during each training epoch. The positional embeddings were derived by randomly perturbing the structure, a process that enables the model to better capture the relative positions of atoms in the system, while also mitigating permutational invariance problems.

#### 2.1.2 Persistence image embeddings

These embeddings are generated from persistent homology images^40^ and encode global structural information about each crystal structure. This complements the local information provided by the atom-based graph embeddings. Persistent homology has shown potential recently in capturing the topological features of porous materials, demonstrating improved performance in adsorption prediction.^41^ More broadly, persistence images in crystal structures encode topological changes as these occur when spheres centered on the atoms increase their radii. These topological changes can include the development of channels (1D persistence image) and voids (2D persistence image) within the structure and can therefore have a critical effect in the global representation of the structure in downstream tasks where geometric information is crucial. As exemplified in Fig. S1,† the existence of channels and voids is encoded in diagrams that are subsequently transformed into images that further capture the spatial distribution of topological features.^40^ Here we segment persistence images of molecular crystals into patches of fixed size and feed them into the transformer encoder as an additional modality. Further details can be found in Section 4. The transformer encoder architecture that we used in our framework was inspired by BERT,^28^ which is based on a bidirectional training strategy employing masked language modeling (MLM) and next sentence prediction (NSP) objectives. A [CLS] token is used to predict desired properties by training a multi-layer perception (MLP) head on it. The subsequent tokens are embeddings of atoms and persistence images are segmented into patches, separated by a [SEP] token. At the end of each image, two [SCL] tokens are added to indicate the maximum persistence value and maximum birth value of the persistence images. This method ensures a more balanced distribution of data across pixels, instead of scaling each image to a universal maximum size, which could lead to the concentration of most of the information within just a few pixels. It also enhances the model's robustness, preventing failures when processing larger-scale images in future applications. The full pre-training architecture is illustrated in Fig. 1b.

### 2.2 Pre-training results

To capture universal latent representations of molecular crystals we designed four pre-training tasks performed on a dataset of 706 126 experimental structures sourced from the Cambridge Structural Database (CSD),^37^ namely a masked atom prediction task (MAP), an atom pair classification task (APC), a crystal density prediction task (CDP) and a symmetry element prediction task (SEP). The MAP and APC tasks capture local chemical information, while the CDP and SEP tasks capture global structure information.

#### 2.2.1 Masked atom prediction (MAP)

**Fig. 2:**
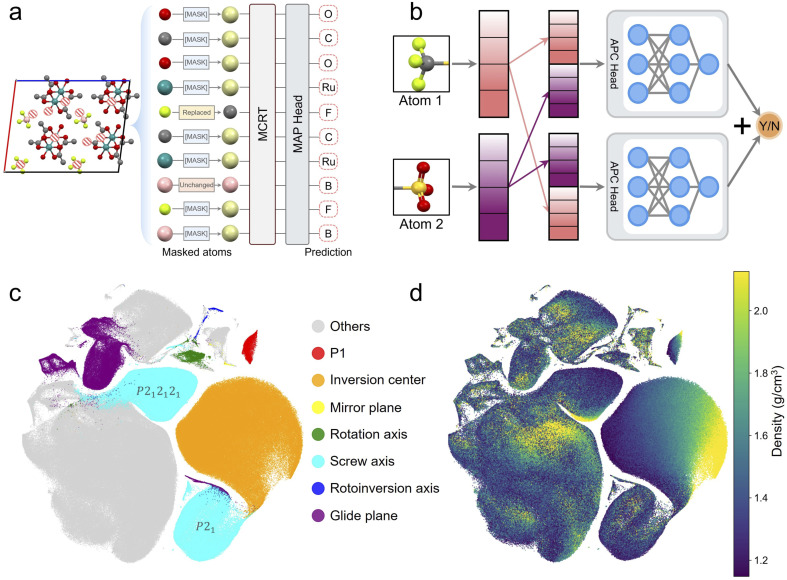
Summary of pre-training tasks. (a) Scheme for masked atom prediction (MAP) task. Among the masked atoms, 80% are replaced with the [MASK] token, 10% are replaced with a random atom, and 10% remain unchanged. (b) Scheme for atom pair classification (APC) prediction head. Representations of a pair of atoms are concatenated in two orders to eliminate the impact of atom sequence, ensuring more stable predictions. (c) The t-SNE embeddings of the [CLS] tokens of 706 126 experimental molecular crystals obtained from the pre-trained model, with crystals containing only one type of symmetry element being coloured. (d) The t-SNE embeddings of the [CLS] tokens of 706 126 experimental molecular crystals obtained from the pre-trained model, with colour indicating density, and the top and bottom 5% of densities truncated for better visualisation.

The goal of the MAP task is to predict the type of randomly selected masked atoms, which gives the model a deeper understanding of the various local chemical environments of atoms. Similar to the masked word prediction task in the BERT model, 15% of the atoms were masked before being inputted into the model. Of these, 80% were replaced with a [MASK] token, 10% were replaced with another random atom, and the remaining 10% were left unchanged (Fig. 2a). This approach avoids replacing all selected atoms with [MASK] tokens as these do not appear in downstream tasks and helps mitigate the mismatch between pre-training and fine-tuning. The accuracy of the MAP task on the pre-training dataset was 99.9%.

#### 2.2.2 Atom pair classification (APC)

In the APC task, the model attempts to distinguish whether a pair of atoms comes from the same molecule. This task is designed to help the model distinguish the different molecules within a crystal cell and to gain a deeper understanding of the crystal structure, noting that intermolecular and intramolecular interactions are highly diverse—more so than for ionic, inorganic crystals. Specifically, for each crystal, a certain number of atom pairs are randomly selected for this process ensuring that half of these pairs come from the same molecule, and the other half from different molecules to balance bias. To ensure that the order of atoms does not affect the prediction results, the representation vectors of the two atoms are concatenated in both forward and reverse order, passed through the same prediction head, and the outputs are summed to obtain the final prediction result, as shown in Fig. 2b. Crystal structures were represented as graphs where disconnected sub-graphs were considered as isolated molecules. If the number of atom pairs is too large, then the training process will be slowed down by the sampling process. Conversely, if the number of pairs is too small, then the training accuracy will improve very slowly. It was observed empirically that using 200 atom pairs per crystal gives a fair balance between training speed and accuracy. The accuracy of APC task on the pre-training dataset was 99.9%.

#### 2.2.3 Crystal density prediction (CDP)

Crystal density is linked to the packing density of molecules and serves as a cheap and easy-to-obtain proxy label during pre-training. Due to the significant impact of molecular packing density on the porosity of molecular crystals, CDP is a particularly important task for applications that depend on crystal voids, such as adsorption, although it might also be expected to have correlations with other solid-state properties, such as charge mobility. Methane deliverable capacity is one example of an adsorption property task, and Fig. S5† illustrates the strong correlation between methane capacity and crystal density for a hydrogen-bonded framework (HOF) forming molecule, T2.^7^ Similar broad correlations would be expected for other gases and other materials. For the prediction of crystal density, the [CLS] token output by the model was passed through a one dense layer head. The mean absolute error (MAE) of CDP on the pre-training dataset was 0.032 g cm^−3^. For reference, 99.6% of crystals in the pre-training set have a physical density of >1 g cm^−3^ (average density = 1.508 g cm^−3^), so this is a relatively small error.

#### 2.2.4 Symmetry element prediction (SEP)

The space group of a crystal can be considered as a blueprint that provides important information about its global structure. However, a direct prediction of space group can be challenging due to the strongly imbalanced distribution of space groups among crystals, where >80% of molecular crystals occupy just 5 of the 230 existing space groups.^15^ This class imbalance, in conjunction with the complex symmetry information contained in underrepresented space groups, often hinders the learning of meaningful space group representations. Instead of using space groups explicitly, we focused on the less imbalanced task of predicting the total symmetry elements that define each space group, which effectively encodes the same foundational information contained in space groups. There are six types of symmetry elements:^42^ inversion center, mirror plane, rotation axis, screw axis, rotoinversion axis, glide plane. Considering the case of no symmetry elements (*P*1 space group), the output of this task then becomes a 7-dimensional multi-hot vector (note that a structure can correspond to multiple different symmetry elements). For a successful prediction, all elements of the prediction must match the label exactly. Pre-training the SEP task on the pre-training dataset results in a prediction accuracy of 98.5%.

To validate the representation learning capacity of our proposed pre-training framework here, we visualised the learned representations by MCRT ([CLS] tokens) of all 706 126 crystals in 2D using t-SNE.^43^ Since a crystal can contain multiple types of symmetry elements, we selected crystals containing only one type of symmetry element and highlighted them with a different colour. The low dimensional map of Fig. 2c validates that crystals with similar symmetry elements cluster together. It is noteworthy that certain symmetry elements manifest as multiple clusters in the t-SNE embeddings, which can be attributed to the nuanced differentiation of space groups. For example, screw axes are present in several space groups, as depicted in Fig. S6.† Specifically, the two prominent clusters correspond to space groups 4 (*P*2_1_) and 19 (*P*2_1_2_1_2_1_). Although the model was not explicitly trained to recognize space group information, it automatically learned and differentiated between various space groups during the pre-training phase. This capability highlights the model's inherent ability to capture and classify structural features of molecular crystals and to comprehend underlying crystallographic principles. Additionally, Fig. 2d shows a re-labeling on the same map using crystal density values, where it can be seen that crystal density exhibits a gradient distribution within most of the symmetry element clusters, indicating that the embedding vectors cluster according to similar densities. Taken together, these results suggest that the pre-trained model has been successfully trained to capture key features of molecular crystals.

### 2.3 Fine-tuning results

Next we demonstrated the utility of our pre-training framework through a series of fine-tuning experiments on a diverse range of crystal property prediction tasks. These include lattice energy prediction, methane deliverable capacity prediction (298 K, pressure cycle of 65–5.8 bar), methane diffusivity prediction (298 K at infinite dilution), bulk modulus prediction, and charge mobility prediction, a task related to organic semiconductors. We also tested MCRT's predictive performance in *Δ-E* tasks; that is, the lattice energy difference between DFT and force field accuracy calculations, to assess its extrapolation to higher accuracy levels of energy prediction.

The datasets used in these tasks spanned a wide variety of molecules. For lattice energy prediction, the dataset included a set of 10 structurally related molecules with small changes in hydrogen bonding functionality, derived from earlier crystal structure prediction (CSP) studies,^6^ providing a relevant test for fine-tuning a model to study a closely related family of molecules. By contrast, the *Δ-E* task involved a deliberately diverse set of 1018 organic molecules, designed to develop a generalised ML model capable of improving lattice energy predictions across broad and chemically diverse areas of molecular space.^15^ The charge mobility predictions focused on 7 pentacene and azapentacene molecules,^10^ which are compounds in organic semiconductor research, while the methane capacity and diffusivity tasks were based on CSP structures for the HOF-forming molecule, T2.^7^ The bulk modulus task involved 25 small, organic molecules selected by farthest-point sampling from the large-scale CSP study.^15^ All datasets (except for *Δ-E*) were randomly split with a train-validation-test ration of 80% : 10% : 10%. The *Δ-E* dataset was split according to the original paper.^15^ A detailed description of the datasets and the methods used for their generation can be found in Section 4.

Table 1 reports the mean absolute error (MAE) results for fine-tuning MCRT and its variants, compared against state-of-the-art baseline models. SOAP-based random forest (RF)^44^ and kernel ridge regression (KRR),^45^ graph-based CGCNN^13^ and ALIGNN,^39^ and pre-trained crystal twins (CT)^36^ were selected as baseline models due to their universality and competitive performance in predicting materials' properties.^12,41,46^ For a detailed description of these methods and their featurisations, as well as the MCRT variants used in benchmarking, see Section 4.

**Table 1:** Mean absolute error (MAE) results for the fine-tuned MCRT models and baseline models for a wide range of properties

Property (size, unit)	RF	KRR	CGCNN	ALIGNN	CT	MCRTp^f^	MCRTi^g^	MCRTa^h^	MCRT
LE_all (70k, kJ mol^−1^)^a^	7.79	6.90	5.95	2.68	4.85	3.31	2.63	2.59	**2.34**
LE_T2 (8k, kJ mol^−1^)^a^	7.79	8.44	6.13	3.45	5.21	3.84	3.20	3.27	**2.96**
LE_T2A (1k, kJ mol^−1^)^a^	3.34	3.96	3.20	3.27	3.19	2.98	2.60	2.57	**2.15**
MC (5k, v STP/v)^b^	11.60	11.75	15.87	12.17	14.53	9.88	10.81	9.91	**8.82**
MD (5k, 10^−8^ cm^2^ s^−1^)^b^	0.75	0.68	1.08	0.79	0.92	0.50	0.57	0.48	**0.42**
CM (1k, cm^2^ V^−1^ s^−1^)^c^	0.59	0.60	0.70	0.62	0.62	0.62	0.54	0.60	**0.52**
BM (6k, GPa)^d^	0.52	0.82	0.59	0.56	0.58	0.65	0.56	0.60	**0.51**
Δ-*E* (11k, kJ mol^−1^)^e^	2.82	3.13	2.33	2.90	2.25	1.82	1.62	1.70	**1.57**

a LE_all (70k), LE_T2 (8k) and LE_T2A (1k) denote lattice energy with 73 779, 8293 and 1367 data points, respectively.

b MC (5k) and MD (5k) denote methane capacity and methane diffusivity with 5687 data points, respectively.

c CM (1k) denotes charge mobility with 1130 data points.

d BM (6k) denotes bulk modulus with 6087 data points.

e Δ-*E* (11k) denotes the difference in lattice energy between DFT and force field calculations, comprising 11 458 data points.

f MCRTp is MCRT without pre-training.

g MCRTi is MCRT without persistence image part.

h MCRTa is MCRT using absolute positional embedding. The best results for each property are highlighted in bold.

For lattice energy prediction, three datasets of different sizes were used to validate the model's transferability capabilities under limited data availability scenarios. We note here that LE_all includes the CSP landscapes of 10 molecules, all with hydrogen bonding functionality (see Fig. 5a, below), comprising 73 779 structures in total with their associated lattice energies. LE_T2 represents the CSP landscape of the T2 molecule with 8293 structures and energies, while LE_T2A corresponds to the CSP landscape of the T2A molecule with 1367 structures and energies. Both LE_T2 and LE_T2A are subsets of LE_all.

The fine-tuned MCRT model outperformed all other models across all tasks, demonstrating both superior predictive capability and universality. ALIGNN exhibited better performance compared to other baseline models when predicting LE_all (70k) and LE_T2 (8k), but its performance on LE_T2A (1k) does not stand out against other models. We hypothesise that this could be due to ALIGNN encoding angular information, and thus making the model more complex than other baseline models and more prone to overfitting with insufficient training samples. By contrast, MCRT, with proper pre-training, still demonstrates relatively good predictive performance even with this small dataset of 1367 structures and energies. Graph-based models outperform SOAP descriptor-based models in predicting lattice energy, a property strongly related to the local chemical environment of atoms. Deep models on the other hand perform poorly in predicting methane capacity (MC) and diffusivity (MD) which are properties related to global structural features. A similar observation was also confirmed by studies using the MOFTransformer model.^24^ This phenomenon is further validated by the poorer performance of the purely graph-based MCRTi model. When the persistence image component is added, the MCRT model's performance improves significantly, further emphasising the importance of global geometric features in adsorption and diffusion predictions. Regarding the performance of the non-pre-trained MCRTp, it maintains a competitive performance, but is noticeably inferior to the pre-trained MCRT. For predicting bulk modulus, non-pre-trained MCRTp performed worse than the descriptor-based RF model and graph-based models. However, after pre-training, MCRT achieved the highest predictive accuracy, demonstrating the effectiveness of the pre-training strategy. The ablation model MCRTa used absolute positional embeddings as opposed to relative ones. Despite undergoing the same pre-training stage, its prediction accuracy across various tasks was consistently inferior to that of MCRT, indicating that absolute positional embedding, which does not satisfy translational and rotational invariance, indeed increases training difficulty.

**Fig. 3:**
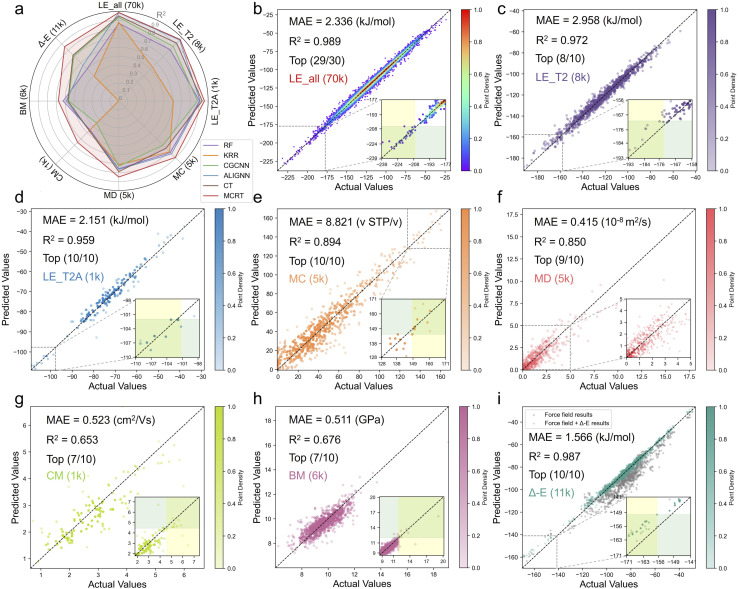
MCRT outperforms other baseline models for all downstream prediction tasks, identifying the top few structures of interest. (a) The coefficient of determination *R*^2^ on test sets of MCRT and baseline models. The prediction results on test sets of fine-tuned MCRT for (b) LE_all (70k), (c) LE_T2 (8k) (d) LE_T2A (1k), (e) MC (5k), (f) MD (5k), (g) CM (1k), (h) BM (6k), (i), *Δ-E* (11k). Inset sub-figures are illustrations of areas of interest, the yellow regions represent the areas where the top *n* actual values are located, while the green regions indicate the areas where the top *n* predicted values are located. The points in the intersections represent the top n points that were successfully predicted. For MD (5k), the sub-figure is intended to provide a clearer visualisation of the densely populated region.

Fig. 3 reports test-set results in *R*^2^ for MCRT and baseline models along with performance correlation plots of MCRT across all downstream prediction tasks. From the radar plot in Fig. 3a, MCRT scores the highest *R*^2^ across all prediction tasks, especially in the data-scarce charge mobility (CM) dataset, where it significantly outperforms other models. Although baseline models may perform well on specific tasks, they struggle to balance performance across diverse tasks. For instance, ALIGNN excels in lattice energy prediction but shows only average performance in other areas. This further highlights the universality of MCRT.

Beyond prediction errors, experimental materials researchers are interested in ranking the best-performing structures to prioritize as experimental targets. For lattice energy predictions, structures with lower lattice energies are more likely to be synthesizable. Fig. 3b, c, and d, demonstrate that MCRT successfully predicts most of the lowest-energy structures for the molecules considered, highlighting the model's practical utility.

For methane capacity (MC), a property with high computational screening cost, MCRT successfully predicts all the top ten best-performing structures, highlighting its potential for robustly accelerating high-throughput computational screening procedures using crystal structure prediction. Here, this may be a better measure than MAE since we are mainly interested in the best-performing crystals. An extensive analysis is included in Table S2,† where the class of best-performing materials in MC is further expanded to a larger subset, without however affecting MCRT's performance, as opposed to other competitors whose prediction errors deteriorate substantially. Additionally, for methane diffusivity (MD), which is a challenging property to capture and predict using machine-learned interatomic potentials, MCRT still delivered the best predictive performance and successfully identified nine out of the top ten best-performing structures. When it comes to charge mobility (CM), a property with sparse data due to high computational cost, all models struggle somewhat with its prediction, but MCRT still identified seven out of the top ten structures with highest charge mobility. Finally, regarding the *Δ-E* task, MCRT captures more accurately the relative energy relationships across various structures. As shown in Fig. 3i, the corrected energies by MCRT (turquoise points) align more closely with DFT-calculated results than those calculated by force fields (grey points), importantly in the low-energy regime, again successfully predicting all of the top ten structures. Further error analyses for all competitors are provided in Fig. S7.†

### 2.4 Interpretability

**Fig. 4:**
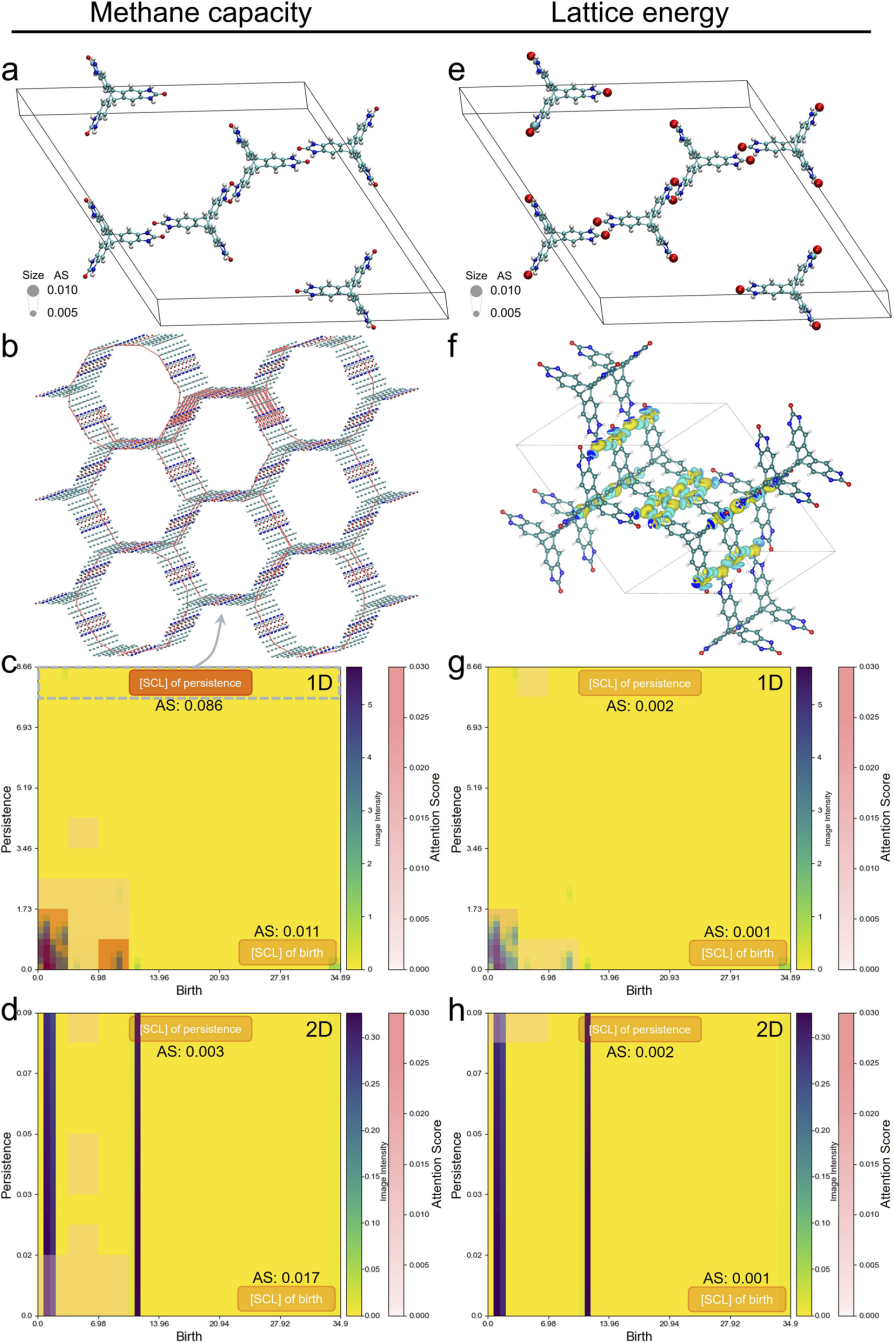
Attention scores for atom-based embeddings and persistence image embeddings for a porous framework, T2-*γ*. (a) Unit cell, (c) 1D and (d) 2D persistence images of T2-*γ* with attention scores for CH_4_ capacity. (b) The point cloud of atoms in T2-*γ*, with red lines representing the representative cycles corresponding to the topological objects in the persistence image. (e) Unit cell, (g) 1D and (h) 2D persistence images of T2-*γ* with attention scores for lattice energy. (f) Electron density difference plot of T2-*γ* highlighting the region of intermolecular interactions (yellow isosurfaces = increased electron density; blue isosurfaces = decreased electron density). The strong intermolecular interactions are found for the atoms that were attended to in e, also for other experimental T2 polymorphs (Fig. S9 and S10†). In the unit cells, the atomic size is proportional to normalized attention scores, with scores less than 0.005 being clipped to avoid extremely small atoms (colour code: C, cyan; H, white; N, blue; O, red). In the persistence images, the 10 patches with the highest attention scores are visualized with a salmon-coloured overlay, where stronger intensities represent higher attention scores.

Feature importance analysis is an inherent capability of transformer models that helps us here to better understand the relationship between molecular crystal structures and their properties. Cumulative attention scores of the [CLS] token were calculated to measure the model's assigned attention to input features according to their importance. An attention rollout strategy^38^ was employed by recursively multiplying attention weights across layers, providing more focused patterns for interpreting which input tokens contribute the most to the model's output. Higher attention scores indicate greater importance for the model's prediction. Fig. 4 provides an intuitive visualisation of the explainability scores for an experimentally synthesized structure, T2-*γ*,^7^ for methane capacity (left column) and lattice energy (right column), illustrating explainable feature importance for both modalities.

When predicting methane capacity, the model shows little attention toward the atomic graph modality (Fig. 4a). However, the model places significantly higher attention on persistence images when predicting methane capacity than when predicting lattice energy (Fig. 4c/g, and d/h), as highlighted by the salmon-coloured areas in the persistence images. This further validates that global geometric features are more important for adsorption predictions, aligning with findings from previous studies.^21,47^ In particular, when predicting methane capacity, the model places particularly high attention to the 1D persistence image, which encodes information about the pores in the crystal. The [SCL] token indicating the largest persistence value is identified as the model's most significant feature, receiving an attention score far exceeding those of other image patches. The persistence value represents the radius of the largest sphere that can pass through the topological object, effectively corresponding to the pore radius. Subsequently we mapped the topological objects within the patches near the largest persistence back to the representative cycles in the original T2-*γ* structure. The objects near the largest persistence value correspond to the large pores in T2-*γ* as shown in Fig. 4b. The largest persistence value thus contains essential information about the pore size, which is crucial for predicting adsorption properties.^48^

For lattice energy predictions, the model exhibits marked attention to the hydrogen-bonding benzimidazole groups of the T2 molecule (Fig. 4e). To evaluate the accuracy of the chemical insights provided by these attention scores, an electron density difference (EDD) analysis was performed on T2-*γ*, as shown in Fig. 4f. The yellow isosurfaces represent regions with increased electron density, while the blue isosurfaces indicate regions with decreased electron density. Normally, a larger magnitude of electron density shift indicates stronger intermolecular interactions. Notably, expanding to a supercell, we observe strong attention in regions corresponding to the areas of intense intermolecular interactions (Fig. S8d†). A similar phenomenon can also be observed in other experimental T2 polymorphs, as shown in Fig. S9 and S10.† This suggests that the model's attention aligns with key regions of strong intermolecular interactions, which play a crucial role in stabilising the crystal structure. Given that subtle changes in these strong interactions can lead to significant differences in lattice energy,^7^ the fine-tuned model appears to have effectively captured the critical chemical features necessary for accurate lattice energy predictions.

### 2.5 Few-shot learning

Data is often the limiting resource both in practical synthetic chemistry and in computational materials studies due to the high costs of synthetic or computational methods. Here we explored our model's extrapolation capabilities on extremely small datasets. Specifically, we tested MCRT's predictive performance in zero- and few-shot learning^49^ scenarios for predicting lattice energies of T2 structures, using datasets containing analogues of T2 to assess its generalization capability within a related molecular family.

**Fig. 5:**
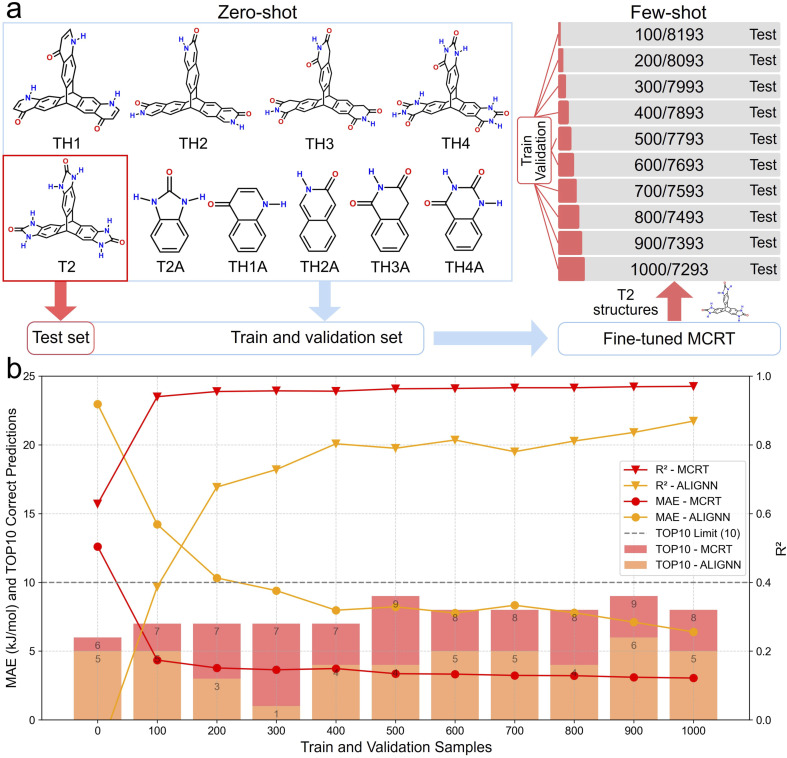
Few-shot learning of lattice energies for 10 related hydrogen-bonding molecules. (a) Chemical structures of T2 and 9 other related hydrogen-bonding molecules, including 4 triptycene framework candidates (TH1–TH4) and 5 small, monoaromatic molecules with the same representative hydrogen bonding functionalities. In the few-shot learning experiment, the T2 structures in the LE_all dataset were extracted and used as the test set. The remaining structures of other 9 molecules were used for training and validation of MCRT, resulting in a fine-tuned MCRT model, yielding the zero-shot prediction results (sampled structures = 0). Subsequently, a small number of T2 structures (100–1000) was randomly sampled as training and validation sets to further fine-tune this model. The remaining T2 structures and associated lattice energies were used as the test set to evaluate the few-shot performance of MCRT. (b) The prediction results of the few-shot learning experiments of MCRT and ALIGNN. Sampled structures refers to the number of T2 structures and associated lattice energies extracted as the training and validation set during few-shot learning scenarios. TOP10 represents the number of correctly predicted structures among the 10 lowest-energy structures.

We first formed a test set using all T2-based structures contained in LE_all (a dataset comprising CSP landscapes of T2 and analogues of T2). The remaining structures were then randomly divided into training and validation sets in a 90%:10% ratio to fine-tune MCRT. Subsequently, from the T2 structures, we sequentially separated 100, 200, 300, …, up to 1000 structures to further fine-tune the model obtained from the previous step. The remaining structures were used as a test set to assess the robustness of MCRT predictions. To provide a clear performance comparison, we conducted the same experiment using ALIGNN, the most competitive baseline model for lattice energy prediction (Table 1). Fig. 5a illustrates our training setup and Fig. 5b presents the MAE, *R*^2^ and top-10 prediction performances for MCRT and ALIGNN. In the zero-shot scenario, MCRT exhibits significantly higher prediction accuracy compared to ALIGNN, indicating that after learning from similar structures, MCRT can generalize more effectively to related but unseen structures. Furthermore, with just an extra step of further fine-tuning on 100 structures, MCRT scores a low prediction MAE (4.34 kJ mol^−1^). By contrast, even after being trained on 10 times more data (*i.e.*, 1000 *vs.* 100 structures), ALIGNN still fails to achieve MCRT's extrapolation capacity, with a prediction MAE of 6.40 kJ mol^−1^. This was further echoed by a series of energy-density landscape reconstruction tasks on the different fine-tuned MCRT models. As shown in Fig. S15,† MCRT accurately reproduces the relative positions of the four experimental porous structures within the energy-density landscape even with zero-shot learning, whereas ALIGNN fails to effectively capture these relative positions, particularly misplacing T2-*δ* by conflating it with numerous other structures, as illustrated in Fig. S16.† This further underscores MCRT's practicality for computationally costly scenarios such as crystal structure prediction.

## 3 Conclusions

We present a new universal transformer model, MCRT, together with a pre-training framework for predicting a wide range of physical properties for molecular crystals. We have designed a multi-modal architecture that has the capacity to comprehensively learn both local and global representations of molecular crystals. This ensures universal transferability for MCRT across different tasks and structures, at least for the tasks attempted here. We tested MCRT's predictive performance by fine-tuning it on various diverse properties such as lattice energy, methane capacity and diffusivity, bulk modulus, as well as charge mobility. Our proposed model both outperformed current state-of-the-art models and showed strong generalisability performance in limited data availability scenarios. This highlights the practical utility in robustly accelerating materials discovery — for example, by rapidly estimating crystal structure prediction energy landscapes based on landscapes calculated for related molecules. Our MCRT model also provides insights into structure–property relationships for molecular crystals through its permeable, interpretable architecture design. While such interpretability should not be equated to causal, physical understanding, it is striking that the attention scores in the MCRT model correlate so strongly with the key intermolecular interactions in lattice-energy prediction tasks, at least for molecules such as T2 that feature dominant hydrogen-bonding patterns (Fig. 4e, f and S9–S11†).

MCRT performs well both in predicting properties across a broad range and in highlighting the top few ‘best-performing’ crystals (Fig. 3b–h). From a practical perspective, both of these tasks are important since there is very often a trade-off between different properties for real applications. To give just one example, to design materials for methane storage, we need to predict structures that have low lattice energies—that is, materials that will be formed in experiments—while having methane capacities that are high enough, rather than simply identifying the hypothetical crystals that absorb the most methane. Both properties, lattice energy and methane capacity, are expensive to calculate. There are other important physical properties relevant to methane storage materials, not investigated here, such as mechanical stability and thermal conductivity, which would add further computational cost to a digital materials screening programme. As such, the development of universal, inexpensive prediction tools is a key priority in computational materials design. We believe that MCRT can serve as a foundational infrastructure for the molecular crystal research community, aiding us in the accelerated exploration of the vast space of molecular crystals.

## 4 Methods

### 4.1 Pre-training datasets

To ensure high-quality crystal structures for the pre-training dataset of MCRT, we selected 706 126 molecular structures from the Cambridge Structural Database (CSD) database,^37^ pre-filtered to satisfy the following criteria: (i) only structures with fully determined three-dimensional coordinates were included to ensure comprehensive spatial information; (ii) only structures with an *R* factor of 0.1 or less were included to ensure high-quality refinement and accuracy of the crystal structures; (iii) structures exhibiting any form of disorder were excluded to avoid complications in subsequent analysis and to maintain data consistency; (iv) only structures without reported errors were included; (v) we excluded polymeric structures, such as metal–organic frameworks, focusing solely on discrete molecular crystals; (vi) only single crystal structures were considered, ensuring higher precision in the determination of atomic positions. This robust pre-filtering process was crucial to ensure the robustness and reliability of our subsequent analysis and training.

### 4.2 Materials analysis

For the manipulation and labeling of the collected pre-training set, we used the Python Materials Genomics (pymatgen) library.^50^ In particular, for the APC task, the crystal structures were represented as graphs, where disconnected sub-graphs were considered as isolated molecules, for the SEP task the space groups of the crystals were first identified and subsequently were mapped to their corresponding symmetry elements. For the remaining tasks the label generation was straightforward using pymatgen. Before being inputted into the model, the crystals were converted into the *P*1 space group to ensure the feasibility of subsequent SEP and APC tasks during pre-training phase. For the persistence image generation, we used MoleculeTDA^21^ to compute persistence images with a resolution of 50 × 50 and a spread of 0.15, consistent with previous studies.^51^ For the t-SNE embedding, we used a perplexity parameter of 50, due to the large size of the pre-training dataset.

### 4.3 Fine-tuning data collection

We fine-tuned MCRT on diverse properties of different molecular crystals to validate the generality of MCRT's predictive capabilities. The details of the datasets are as follows.

#### 4.3.1 Lattice energy

Lattice energy calculations were performed with an anisotropic atom–atom potential using DMACRYS.^52^ Electrostatic interactions were modelled using an atomic multipole description of the molecular charge distribution (up to hexadecapole on all atoms) from the B3LYP/6-311G(d,p)-calculated charge density using a distributed multipole analysis.^53^ Atom–atom repulsion and dispersion interactions were modelled using a revised Williams intermolecular potential,^54^ which has been benchmarked against accurate, experimentally determined lattice energies for a range of molecular crystals.^55^ We specifically generated three fine-tuning datasets of different size to test MCRT's predictive capacity on limited data availability scenarios, namely *LE_all*, a dataset with 73 779 structures composed of CSP landscapes on all the molecules listed in Fig. 5a, *LE_T2*, the CSP landspace of T2 with 8293 structures and *LE_T2A*, the CSP landspace of T2A with 1367 structures.

#### 4.3.2 CH_4_ deliverable capacity

A dataset of 5687 T2-based structures with calculated CH_4_ deliverable capacity (298 K, 65–5.8 bar) was directly retrieved from the previous work.^7^

#### 4.3.3 CH_4_ diffusivity

A dataset of 5687 T2-based structures with calculated CH_4_ diffusion coefficients at infinite dilution using the MD simulations. The simulations were conducted at 298 K with a time step of 1 fs for a total of 5 million cycles, with 1000 cycles used for the initialization and 10 000 cycles for equilibration. DREIDING force field was used with the Lorentz–Berthelot mixing rule and a cut-off distance of 13 Å. The CH_4_ molecule was modeled as a single atom. Prior to the simulations, 30 CH_4_ molecules were randomly introduced into the pores of crystals. The mean square displacement (MSD) of gas molecules during 1–5 ns is used to calculate the diffusion coefficient through Einstein's relation.^53^ All these simulations were carried out at NVT ensemble using RASPA2 package.^56^

#### 4.3.4 Charge mobility

The charge carrier mobility values in this dataset were obtained from previous work^10^ and were calculated using the Marcus theory of charge transport. The dataset is based on crystal structure prediction (CSP) studies, with the studied molecules including pentacene and azapentacenes. The charge carrier mobility calculations were restricted to crystal structures within a 7 kJ mol^−1^ energy range of the global minimum on the energy-density landscapes, capturing low-energy polymorphs most likely to be observed experimentally.

#### 4.3.5 Bulk modulus

Bulk modulus calculations were performed for sets of predicted crystal structures of 25 small, organic molecules from a recent large-scale CSP study.^15^ To include a diverse set of molecules, these were selected by farthest-point sampling of the 1007 molecules from^15^ using Euclidean distances between 1024 bit extended connectivity fingerprints^57^ for evaluating molecular similarity. Calculations were performed for all crystal structures within 12 kJ mol^−1^ of the global energy minimum on each CSP landscape. Bulk moduli were calculated from a Voigt–Reuss–Hill average over elastic stiffness and elastic compliance tensors. Elastic constants were calculated using rigid-molecule calculations in the DMACRYS software^52,58^ with intermolecular interactions modelled using the FIT^59^ exp-6 atom–atom force field and atomic multipole electrostatics from Distributed Multipole Analysis^60^ of B3LYP/6-311G** calculated molecular charge densities. Any crystal structures not satisfying Born stability criteria were removed from the dataset, resulting in a final set of 6087 crystal structures.

#### 4.3.6 Δ-E

The training target, *Δ-E*, represents the lattice energy difference between DFT (B86bPBE + XDM) and force field (FIT + DMA) accuracy. Following the approach in the original paper,^15^ which includes 1000 CSP landscapes, we split the dataset by selecting 10 crystal structures from each of around 900 landscapes for training and validation, while about 100 landscapes, with 10 structures each, were reserved as a test set. An exclusion of duplicate structures was applied that led to a final dataset of 11 458 structures.

### 4.4 Training details

For pre-training, we randomly split the 706 126 molecular crystal dataset with a train-validation ration of 90%:10%. Consistent with BERT_BASE_,^28^ the transformer encoder in MCRT adopts *L* = 12, *H* = 768, and *A* = 12, where *L* represents the number of layers, *H* the hidden size, and *A* the number of self-attention heads. The model was trained for 50 epochs with a batch size of 512. Due to the large pre-training dataset, we selected a relatively large batch size to ensure stable gradient updates. The decision to train for 50 epochs was empirically determined from MCRT's training curves (reported in Fig. S4†), which indicated that the model training converged after 40 epochs. The AdamW optimizer with a learning rate of 10^−4^ and weight decay of 10^−2^ was used.^37^ The learning rate was warmed up during the first 5% of the total epoch and was then linearly decayed to zero for the remaining epochs. For the SEP task we assigned higher weights to the elements with fewer occurrences due to the great variance in the frequency of the occurrence of different symmetry elements. The individual weights are calculated as follows:1
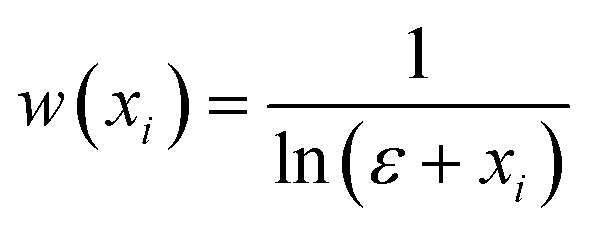
where *w*(*x*_*i*_) is the weight of element *i*, *x*_*i*_ is the frequency of element *i* and ϵ (ϵ ≥ 1) is a parameter to adjust the weight distribution which was set to 1.1 to avoid extremely large weights. The resolution of the persistence image during training was set to 50 × 50 with a patch size of 5 × 5 in accordance to previous studies.^21,51^

For fine-tuning, all datasets (except for *Δ-E*) were randomly split with a train-validation-test ration of 80%:10%:10%. The *Δ-E* dataset was split according to the original paper.^15^ By initializing a single dense layer to the [CLS] token, all model weights are fine-tuned to predict desired properties for 50 epochs with a batch size of 32. All other settings are the same as in the pre-training step.

### 4.5 Baselines and ablations

We test the prediction performance of MCRT against a wide range of baselines and state-of-the-art methods. These include.

– Random forest (RF): A robust ensemble learning algorithm that aggregates the predictions of multiple decision trees, typically leading to enhanced generalisation performance by reducing overfitting and variance in predictive modeling tasks.^44^

– Kernel Ridge Regression (KRR): KRR integrates ridge regression with the kernel trick, enabling it to perform nonlinear regression in high-dimensional feature spaces while controlling for model complexity through regularisation.^45^

– Crystal Graph Convolutional Neural Network (CGCNN): CGCNN represents crystalline materials as graphs, where atoms serve as nodes and bonds as edges, and learns material properties by applying convolutional operations over the graphs.^13^

– Atomistic Line Graph Neural Network (ALIGNN): ALIGNN enhances conventional graph neural networks by incorporating bond angle information from line graphs, thereby improving the model's capability to predict complex material properties with higher accuracy.^39^

– Crystal Twins (CT): CT is a self-supervised pre-trained model for crystalline material property prediction, using twin CGCNNs to learn robust representations from large unlabeled datasets, which are then fine-tuned for specific tasks.^36^

For descriptor-based models, the Smooth Overlap of Atomic Positions (SOAP) descriptor was used due to its universality.^16^ The parameters for the SOAP descriptor were set as follows: a cutoff for the local region of 4.0 Å, 6 radial basis functions, and a maximum degree of spherical harmonics of 6. RF and KRR implemented in scikit-learn^61^ were adopted, and the hyperparameters were tuned using grid search. For RF, the number of trees was searched from 10 to 1000. For KRR, the regularization strength *ω* was searched from 0.001 to 100. For Graph Neural Networks (GNNs), CGCNN was trained with the following hyperparameters: 32 batch size, 100 epochs, 5 message passing layers, 1 hidden layer after pooling, 64 hidden atom features in message passing layers. ALIGNN was trained with the following hyperparameters: 32 batch size, 100 epochs, 4 message passing layers, 1 hidden layer after pooling, 256 hidden atom features in message passing layers, in line with the original paper.^39^ For the crystal twins pre-trained model (CT), the same fine-tuning hyperparameters as the original paper were used (128 batch size, 200 epochs, 3 message passing layers).^36^

Additionally, the following variants of MCRT were included in the fine-tuning comparisons to assess the importance of the different learning components of the proposed framework.

– MCRTp: The complete architecture of MCRT used directly for prediction without pre-training.

– MCRTi: The architecture of MCRT without persistence image modality input module.

– MCRTa: The architecture of MCRT using absolute positional embeddings instead of relative ones.

Both MCRTi and MCRTa underwent the same pre-training process as MCRT. For the latter, the absolute positional input features are processed as in ref. 27, that is by employing BERT's native positional embedding module to embed each of the three-dimensional atomic coordinates separately, and eventually summing them up.

### 4.6 The electron density difference analysis

Periodic DFT calculations, including the electron density calculation, were carried out within the plane-wave pseudopotential formalism, using the Vienna *ab initio* simulation package (VASP) code version 5.4.4.^62^ Projector augmented-wave (PAW) method was applied to describe the electron-ion interactions.^63^ Generalized gradient approximation (GGA) with the Perdew–Burke–Ernzerhof (PBE) exchange-correlation functional was adopted to treat electron interaction energy.^64^ Grimme's semi-empirical DFT-D3 scheme with Becke–Johnson damping functions was used here to give a better description of interactions.^65–67^ A kinetic-energy cut-off of 600 eV was used to define the plane-wave basis set. The electronic Brillouin zone was integrated with the smallest allowed spacing between *k*-points (KSPACING) being 0.4 Å^−1^, and the generated grid was centered at the *Γ*-point. The convergence threshold for self-consistency was set to 10^−6^ eV during total energy and force calculations.

The electron density difference (EDD) plots were generated by subtracting the electron densities of each isolated molecule from the electron density of the entire crystal:2
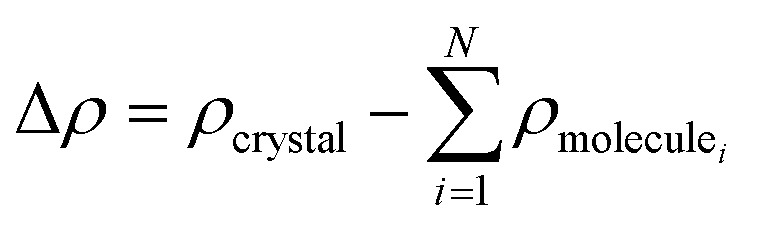
where *ρ*_crystal_ is the electron density of the crystal, and *ρ*_molecule_*i*__ represents the electron density of the *i*-th isolated molecule in the crystal.

## Author contributions

M. F, C. Z and X. E conceived the project, and X. E, G. M. D and A. I. C supervised the project. M. F and C. Z performed the computational experiments and X. E, G. M. D and A. I. C analysed the output of the experiments. All authors contributed to writing the manuscript.

## Conflicts of interest

The authors declare no competing interests.

## Supplementary Material

SC-OLF-D5SC00677E-s001

## Data Availability

Source data and datasets used in this work, including reference codes of molecular crystals screened from the CSD, are available *via* Figshare at https://doi.org/10.6084/m9.figshare.27844302. Additionally, we provide pre-trained MCRT model and fine-tuned versions for all datasets, accessible *via* Figshare at https://doi.org/10.6084/m9.figshare.27822705. The MCRT library is available at https://github.com/fmggggg/MCRT. For ease of use, pre-defined Apptainer images are available on Figshare at https://doi.org/10.6084/m9.figshare.26390275. To ensure reproducibility, all results in this paper are obtained from version 1.0.2 of the MCRT library, which is available at https://pypi.org/project/MCRT-tools/1.0.2.
