# Supplementary material for: A universal foundation model for transfer learning in molecular crystals
Source: Chem Sci. 2025 May 21;16(28):12844–59. doi: 10.1039/d5sc00677e (PMC12174991; doi:10.1039/d5sc00677e)
Supplement: SC-016-D5SC00677E-s001 [file SC-016-D5SC00677E-s001.pdf]

Supplementary Information (SI) for Chemical Science.
This journal is © The Royal Society of Chemistry 2025

Supplementary Information

A Universal Foundation Model for Transfer Learning in Molecular Crystals

Minggao Feng[1], Chengxi Zhao[1], Graeme M. Day[2*], Xenophon Evangelopoulos[1,3*] and Andrew I. Cooper[1,3*]

[1]Materials Innovation Factory and Department of Chemistry, University of Liverpool, Liverpool, UK.
[2]School of Chemistry and Chemical Engineering, University of Southampton, Southampton, UK.
[3]Leverhulme Research Centre for Functional Materials Design, Liverpool, UK.
*Corresponding author(s). E-mail(s): g.m.day@soton.ac.uk, evangx@liverpool.ac.uk, aicooper@liverpool.ac.uk

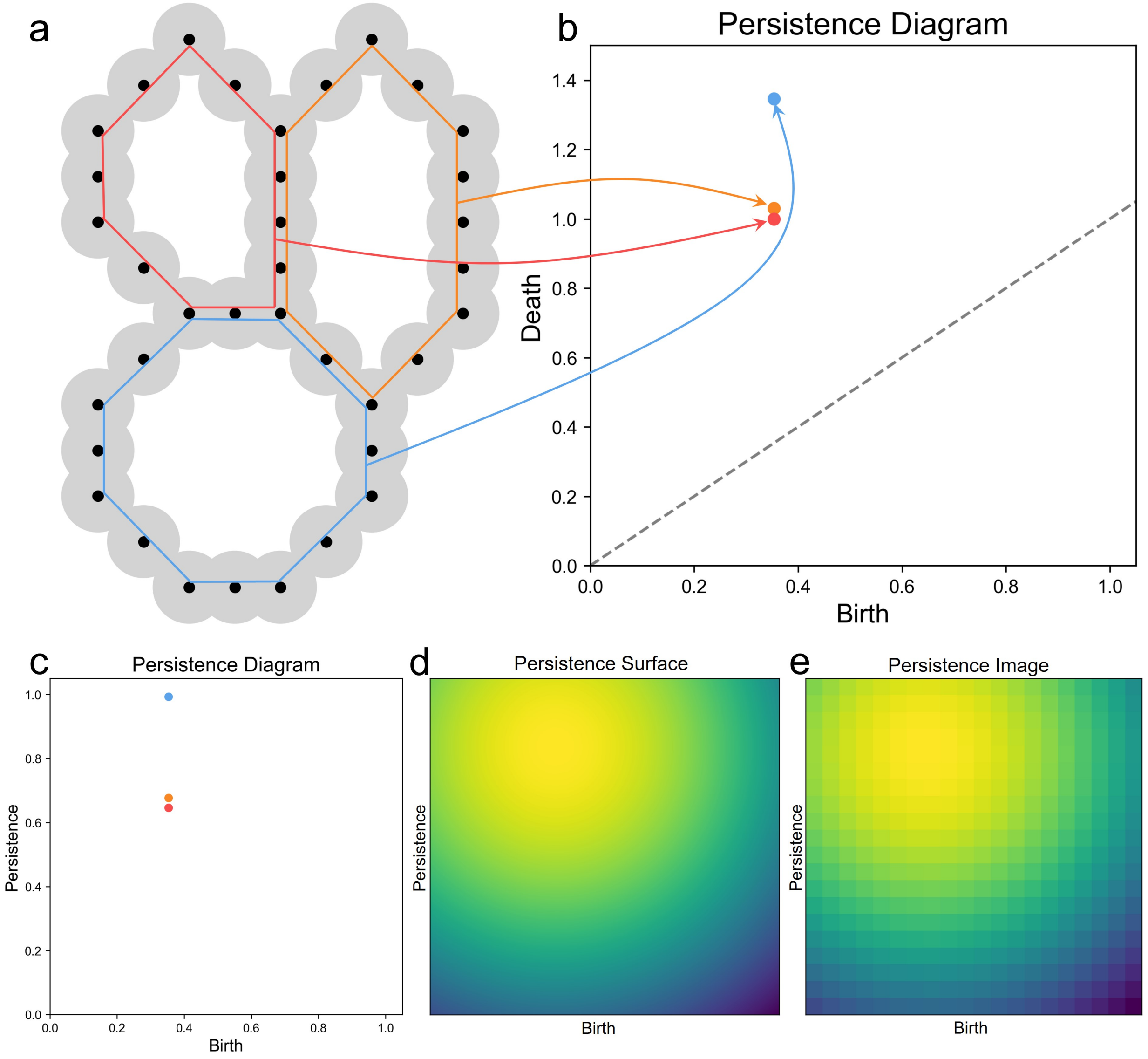

Fig. S1 Scheme outlining mapping of point cloud to persistence image. a, A point set (representing atomic centers) with balls of increasing radius around the points. **b**, 1-Dimensional persistence diagram of the point set in birth-death coordinates. Representative cycles, corresponding to the points in the diagram, are highlighted with matching colours. **c**, 1-Dimensional persistence diagram of the point set in birth-persistence coordinates. **d**, The corresponding persistence surface is defined as a weighted sum of Gaussian functions, with each Gaussian centered at a point in the PD. **e**, The corresponding persistence image is obtained by converting the surface into a finite-dimensional vector, which is achieved by discretising a relevant subdomain and then integrating the surface values over each discrete region. The set resolution of the persistence images is 50 × 50, which is subsequently partitioned into 5 × 5 patches for transformer input.

Persistence images

The process of generating persistence images from point clouds is simplified into a two-dimensional representation, as illustrated in Fig. S1. The molecular crystal is represented as a union of spheres centered on atomic positions, with radii systematically increased to track changes in the topology of their union. These changes, which correspond to the appearance or disappearance of features like loops and voids, are recorded as birth–death pairs in persistence diagrams. The birth value represents the scale at which a topological object appears, and the death value represents when it disappears, with the difference defining the persistence of the topological object. Persistence diagrams, computed using the Dionysus library (https://github.com/mrzv/dionysus), are transformed into birth–persistence pairs to emphasize the stability and prominence of features. These pairs are then convolved with Gaussian functions, smoothed, and discretized onto a fixed-resolution grid to create persistence images. This approach translates the structural and topological information of the crystal into a standardized vector format suitable for machine learning, enabling direct comparison across structures and efficient extraction of meaningful features.

To normalize the size of molecular crystals, we fill a (100 Å)^3 super cell with the atoms of the crystal. The size is large enough to capture the statistics of the distribution of the topological features in every structure. The resolution of persistence image is 50 × 50 and the Gaussian spread is 0.15.

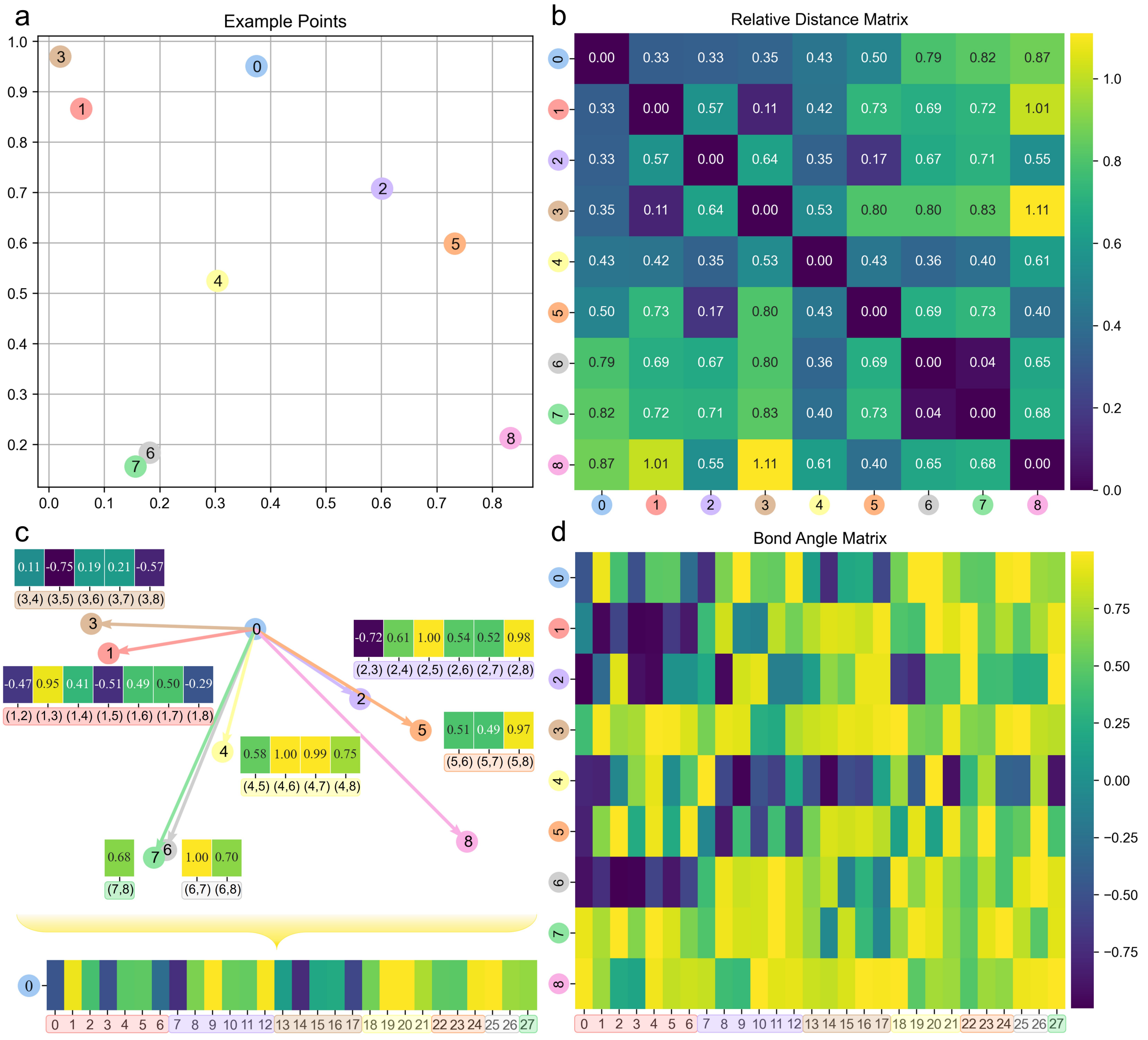

Fig. S2 Scheme of relative positional embedding. **a**, Example points with randomly generated coordinates. **b**, Relative distance matrix of the example points. **c**, Scheme showing the generation of bond angle vector of point 0. The order of angles is based on the distances between point 0 and neighbour points. **d**, Bond angle matrix of the example points.

Relative postional embedding

The relative positional embedding, in contrast to absolute positional embedding that uses 3D coordinates, employs a relative distance matrix and a bond angle matrix to generate positional embedding. These two matrices are individually processed through separate multi-layer perceptrons (MLPs) to match the dimensionality of the atomic representation and are then directly added to the atomic representation. The relative distance matrix provides information about the distances between each atom and all other atoms, while the arrangement of bond angles in the bond angle matrix is based on the order of atomic distances. This enables the model to deduce the relative positions of atoms from these matrices. It is important to note that the use of relative positional embedding achieves translational and rotational invariance, capabilities not possible with absolute positional embedding.

The calculation of the relative distance matrix is straightforward, but the computation of the bond angle matrix requires careful design. As shown in Fig. S2c and d, we calculate and organize the angles formed between a central atom and its surrounding atoms based on their distances. Starting from the closest neighbors, we sort the surrounding atoms by their distance to the central atom. For each neighbor, we progressively calculate the angles between its vector and the vectors of other, more distant neighbors. This approach results in a structured matrix of angles, ordered by distance, which captures the spatial relationships around the central atom.

In crystal or molecular structures, multiple atoms may be equidistant from a central atom, especially in symmetric structures. These equidistant atoms are grouped to capture local geometric features and improve computational efficiency, while also ensuring that angle representations are unique and unaffected by the order of atoms within the same distance group. To accurately capture the angular relationships within each group, we calculate a squared mean of the angles between atoms in the group as the internal average angle of this group, introducing a nonlinearity that enhances the distinction between different angular arrangements compared to a simple average. We then calculate the central vector by summing the unit vectors of each atom in the group, ensuring that the resulting vector maintains rotational equivariance. In subsequent angle calculations, when two neighboring atoms belong to the same group, the internal average angle of that group is used. When only one neighboring atom belongs to a group, the central vector of that group is used to calculate the angle with the other atom. This approach reduces computational complexity and preserves the overall directional information, while also ensuring rotational invariance and uniqueness in the encoding. To capture all the possible bond angles, the number of neighboring atoms is set to 8.

To demonstrate the practical utility of using relative positional embeddings in the architecture of MCRT, we compare its pre-training performance against a variant without relative positional embeddings (MCRT-wope). Fig. S4 highlights the importance of relative positional embeddings in better capturing both local and global structural information essential for the APC and SEP tasks.

Top-performing structures prediction for methane capacity

For practical methane storage applications, the ability to accurately predict high-performing materials is critical. One may argue that lower MAEs might simply indicate that MCRT is more accurate for average materials, while its performance for the more practically relevant top-performing materials could be suboptimal. To address this concern, we conducted further analysis on the methane deliverable capacity dataset. As shown in Fig. S3, the distribution from the random split reveals that values above 80 v STP/v represent the tail of top-performing materials. Based on this observation, we set 80 v STP/v as a threshold and separately evaluated the prediction performance (MAEs) for both average and top-performing materials. As detailed in Table S1, our MCRT model not only performs well on the entire dataset but also achieves even lower MAEs for the top-performing subset. In contrast, some models, such as CGCNN, exhibit a significant decline in performance on the >80 v STP/v test set. This analysis indicates that while certain models excel in regions with abundant training data, MCRT is capable of accurately capturing the full range of methane capacity—even with fewer high-end training samples—thereby underscoring its practical relevance.

Table S1 Mean absolute error (MAE) results for methane deliverable capacity across different performance ranges.

	RF	KRR	CGCNN	ALIGNN	CT	MCRT
>80 structures (v STP/v)	12.055	13.459	26.347	15.653	18.564	8.713
<80 structures (v STP/v)	11.713	11.371	13.557	11.314	12.265	8.844
Whole dataset (v STP/v)	11.750	11.600	15.874	12.750	14.529	8.821

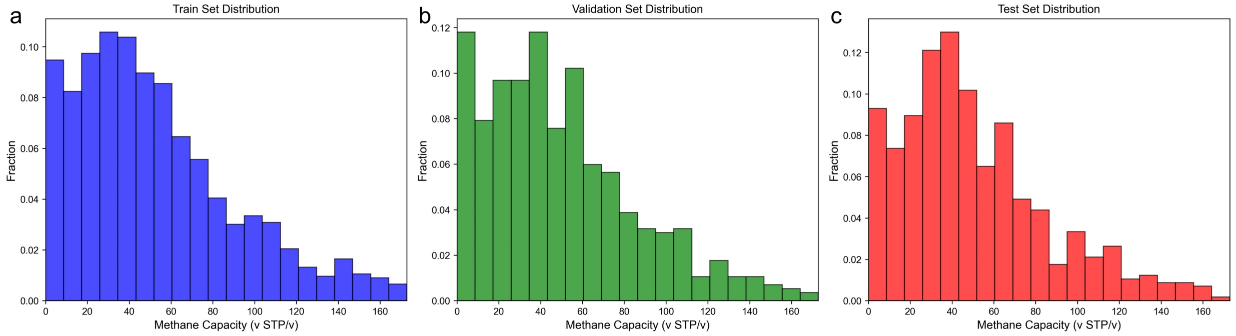

Fig. S3 Data distribution of the methane capacity dataset. The data distribution of **a**, train set, **b**, validation set and **c**, test set.

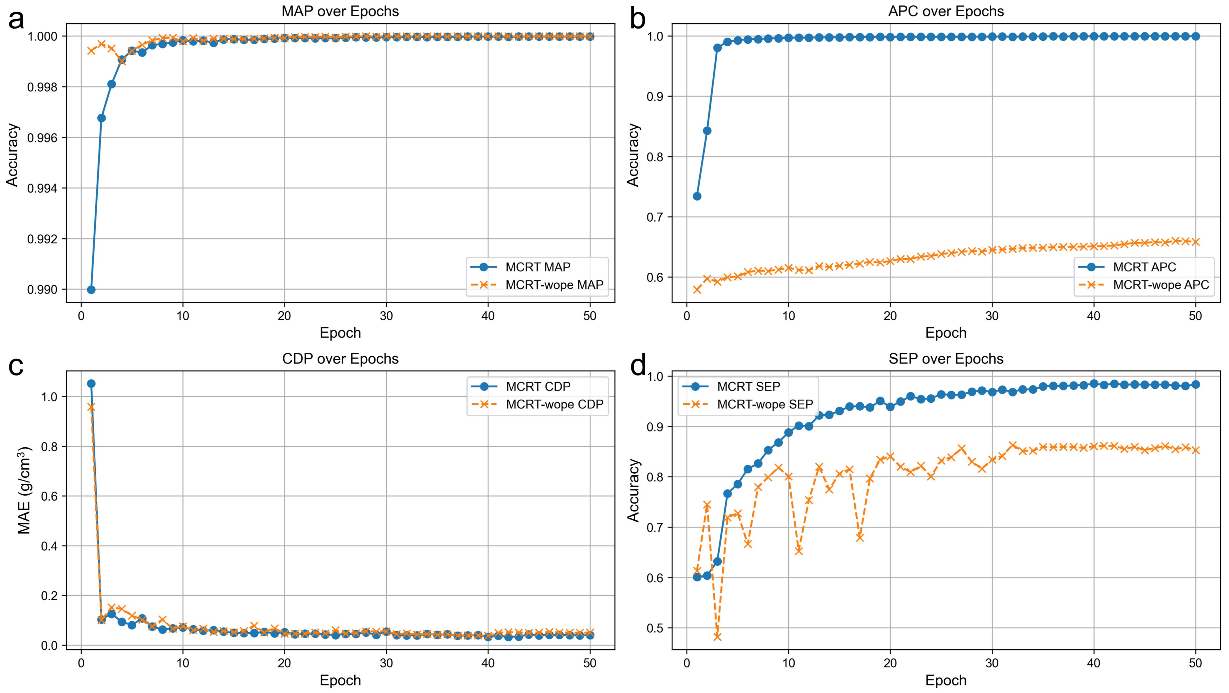

Fig. S4 Pre-training curve of MCRT and MCRT-wope. MCRT-wope denotes MCRT without positional embeddings and evidently fails to capture structural information for the APC and SEP pre-training tasks.

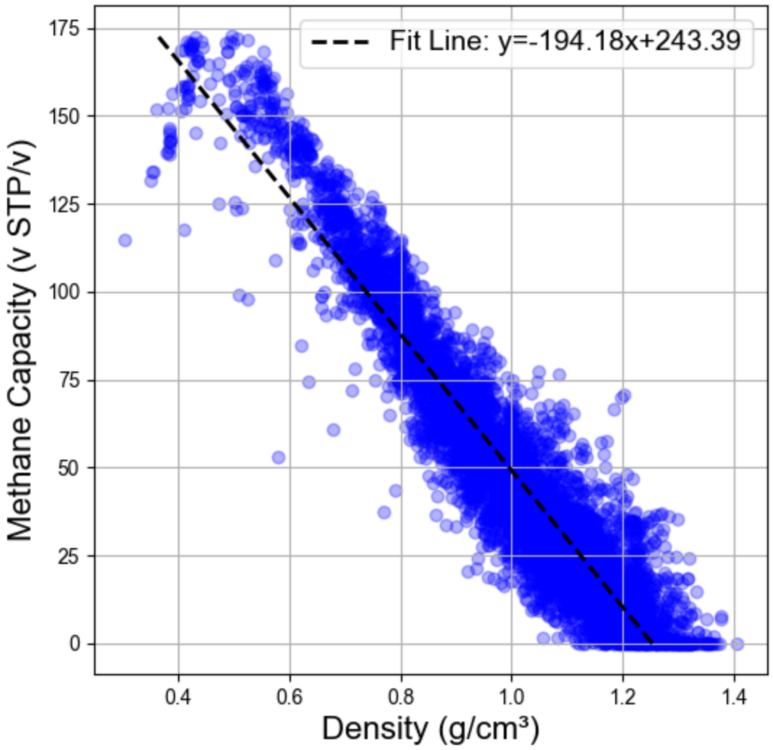

Fig. S5 Correlation plot of methane deliverable capacity vs. density for T2 structures (*Nature* **543**, 657 (2017)). A similar broad correlation between density, which is inversely proportional to pore volume, and gas storage capacity might be expected for other gases and other frameworks.

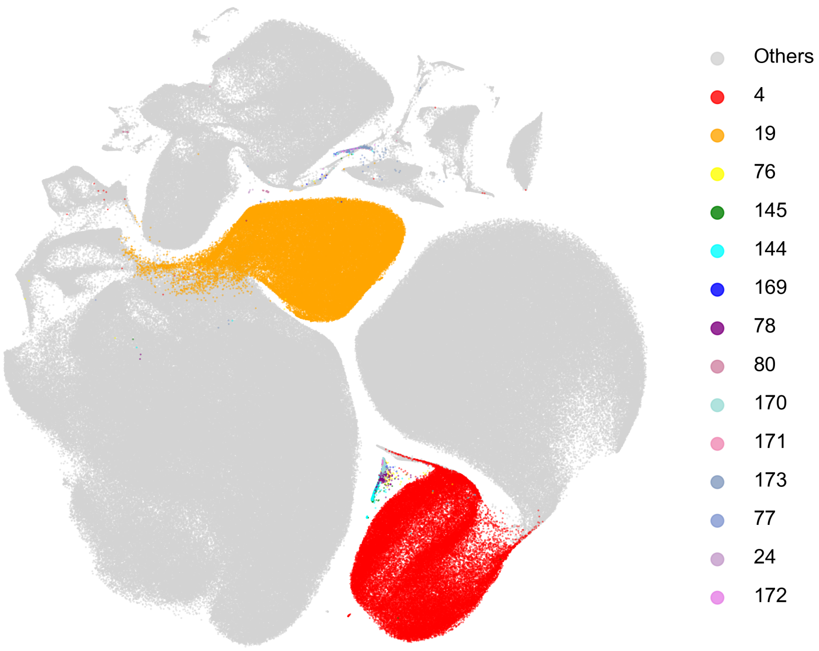

Fig. S6 The t-SNE embeddings of the [CLS] tokens of 706,126 experimental molecular crystals. Obtained from the pre-trained model, with crystals containing only screw axis being coloured by space groups.

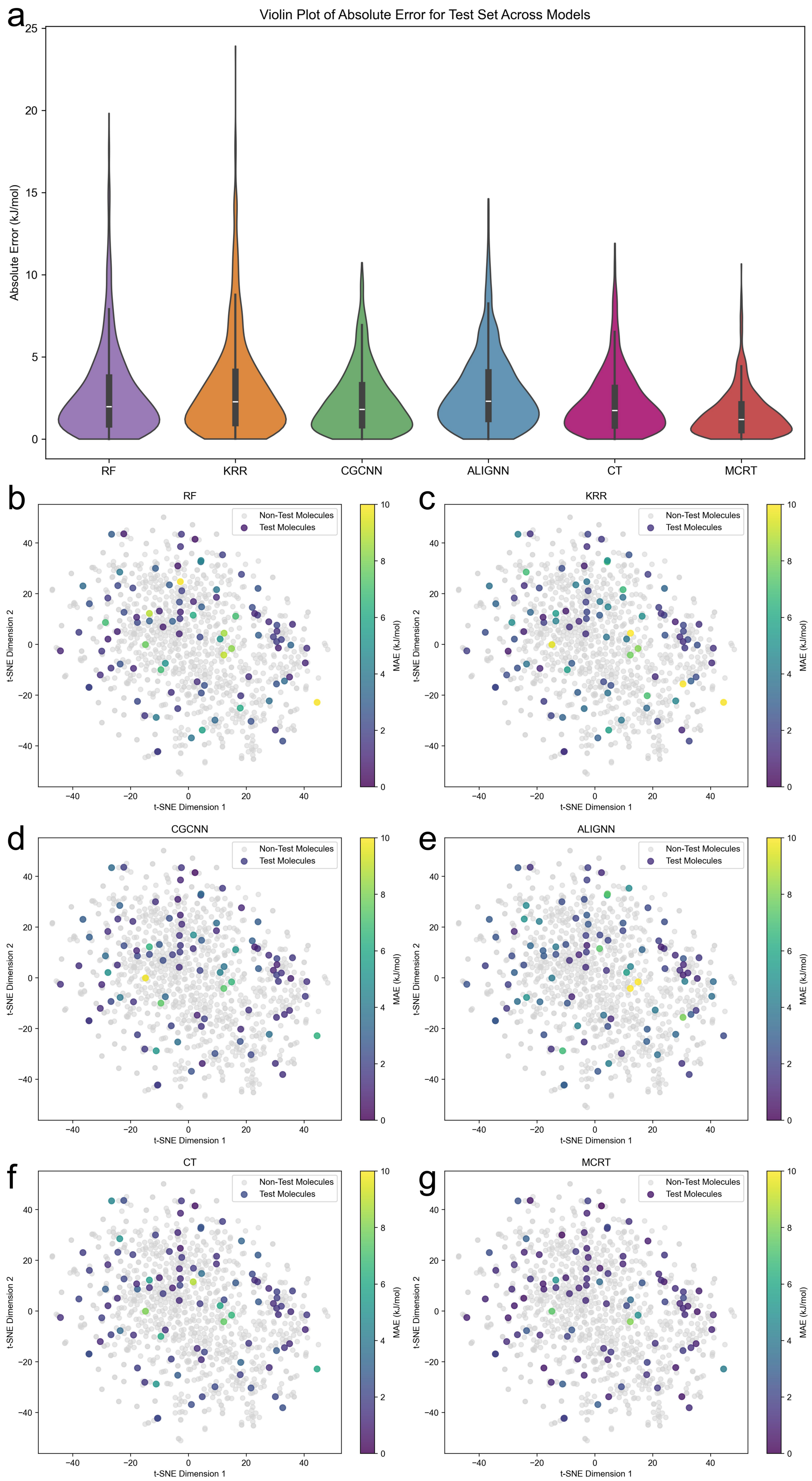

Fig. S7 Test-set results for MCRT and baseline models for the Δ-E task. a, The violin plot showing the absolute errors on the test set for the Δ-E task across MCRT and baseline models. **b**–**g**, The t-SNE embedding of extended connectivity fingerprints (ECFPs) for molecules in the Δ-E task, with colors representing the MAE of each molecule in the test set. MAEs larger than 10 kJ/mol were capped.

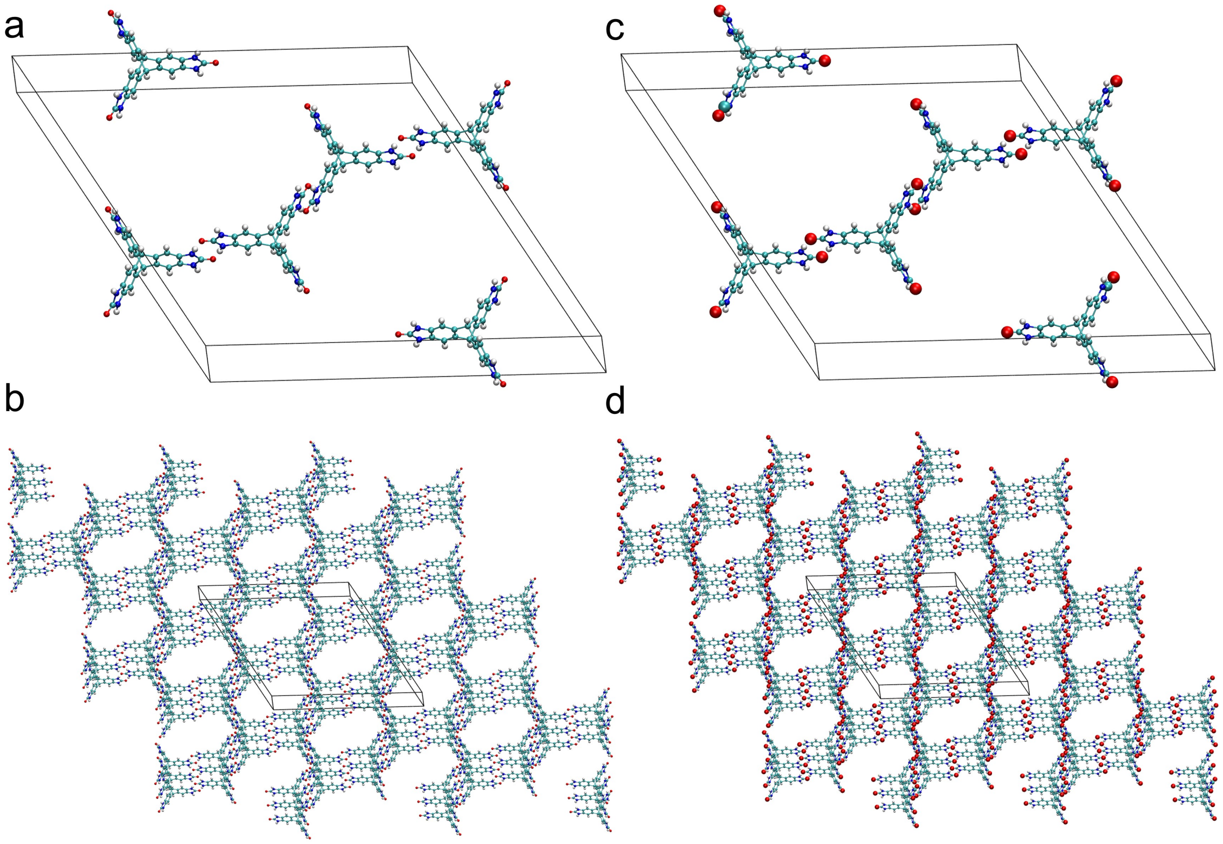

Fig. S8 Attention scores for atom-based embeddings in T2-γ. **a**, Unit cell and **b**, Supercell of T2-γ with attention scores from fine-tuned MCRT that predicts CH_4 capacity. **c**, Unit cell and **d**, Supercell of T2-γ with attention scores from fine-tuned MCRT that predicts lattice energy. The atomic size is proportional to normalized attention scores, with scores less than 0.5 being clipped to avoid extremely small atoms (colour code: C, cyan; H, white; N, blue; O, red).

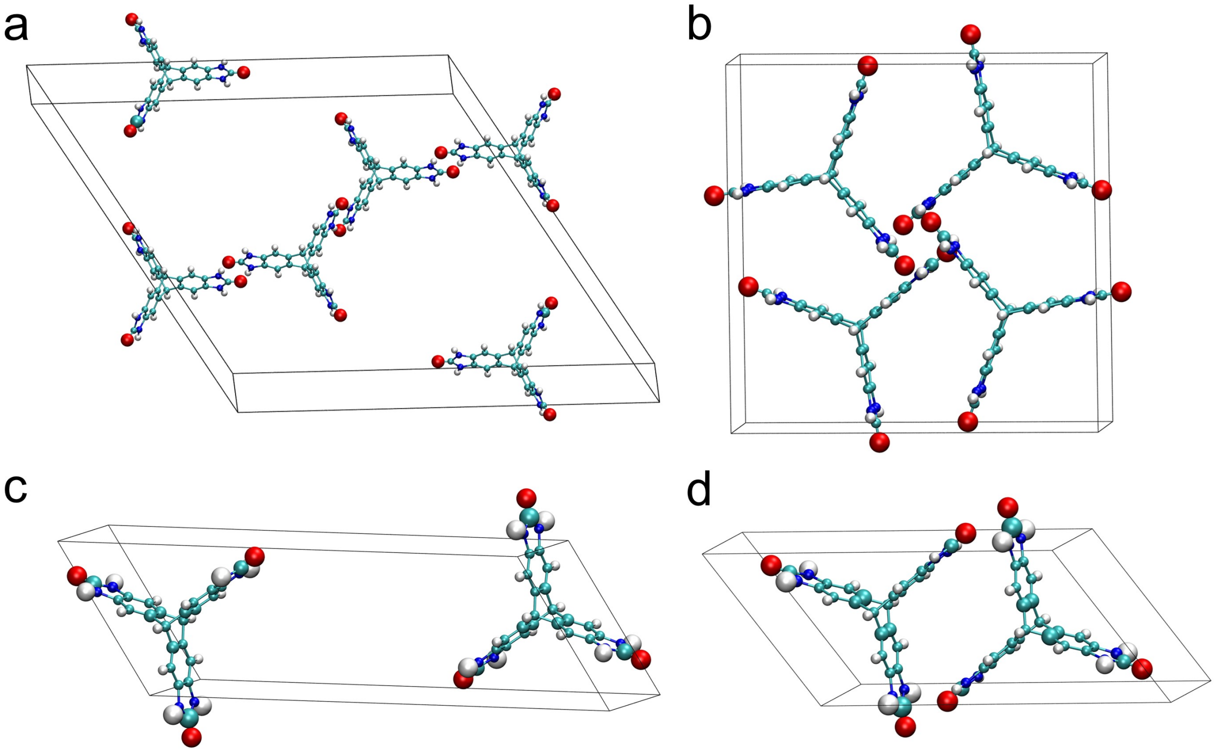

Fig. S9 Attention scores for atom-based embeddings in unit cells of experimental T2 structures. Unit cell of **a**, T2-γ **b**, T2-α **c**, T2-β and **d**, T2-δ with attention scores from fine-tuned MCRT that predicts lattice energy. The atomic size is proportional to normalized attention scores, with scores less than 0.5 being clipped to avoid extremely small atoms (colour code: C, cyan; H, white; N, blue; O, red). For all four of these T2 polymorphs, the atoms involved in hydrogen bonding are the most attended to by the MRCT model. This agrees with experiment, since all four of these polymorphs are dictated by hydrogen bonded 1-D tapes that run parallel to the 1-D pores (*Nature* **543**, 657 (2017)).

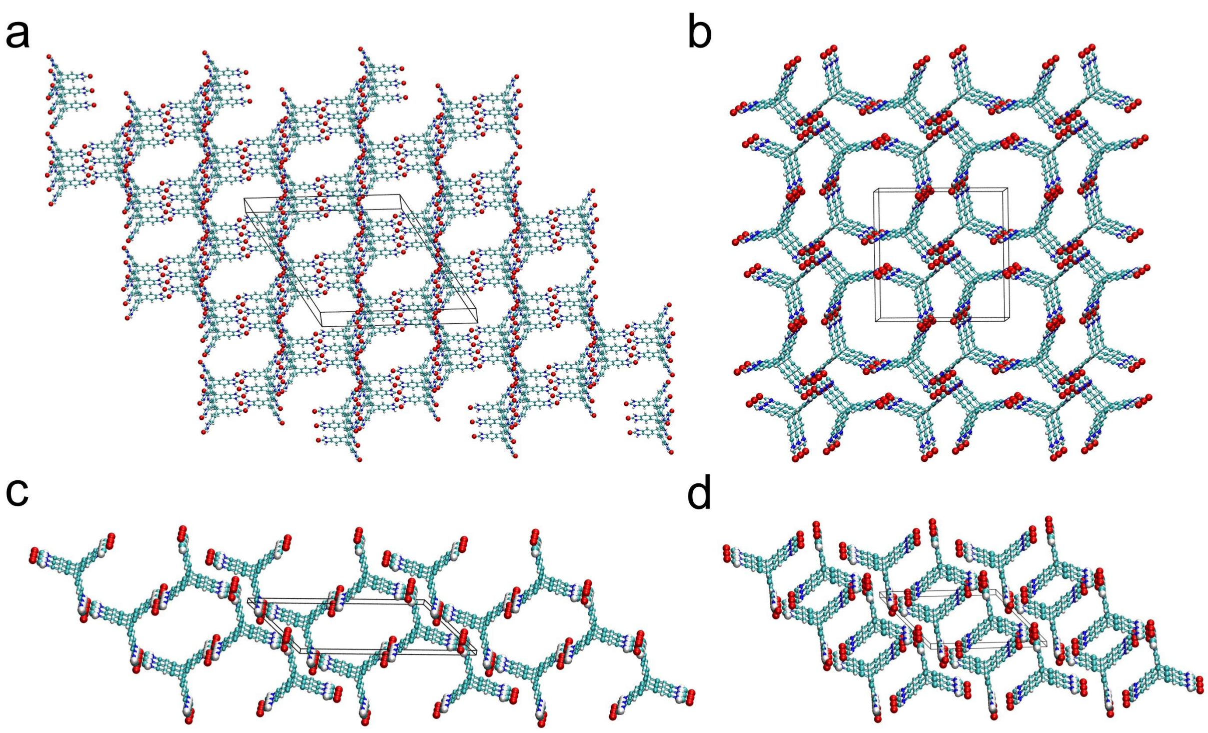

Fig. S10 Attention scores for atom-based embeddings in supercells of experimental T2 structures. Supercell of **a**, T2-γ **b**, T2-α **c**, T2-β and **d**, T2-δ with attention scores from fine-tuned MCRT that predicts lattice energy. The atomic size is proportional to normalized attention scores, with scores less than 0.5 being clipped to avoid extremely small atoms (colour code: C, cyan; H, white; N, blue; O, red).

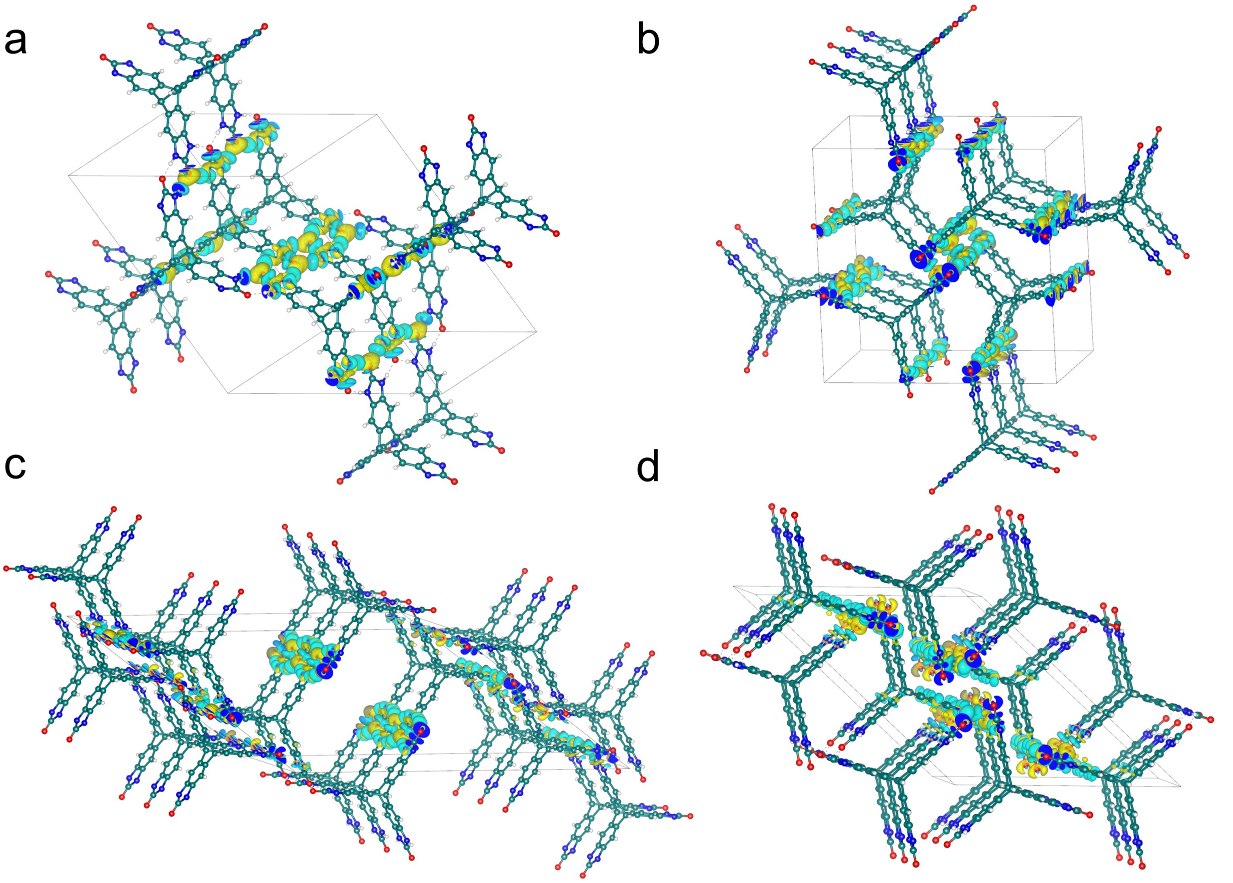

Fig. S11 Electron density difference (EDD) plots. **a**, T2-γ, **b**, T2-α, **c**, T2-β and **d**, T2-δ highlighting the region of intermolecular interactions. The yellow isosurfaces represent regions with increased electron density, while the blue isosurfaces indicate regions with decreased electron density. These areas of strong intermolecular interaction correspond to the atoms attended to in Fig. S9 and Fig. S10.

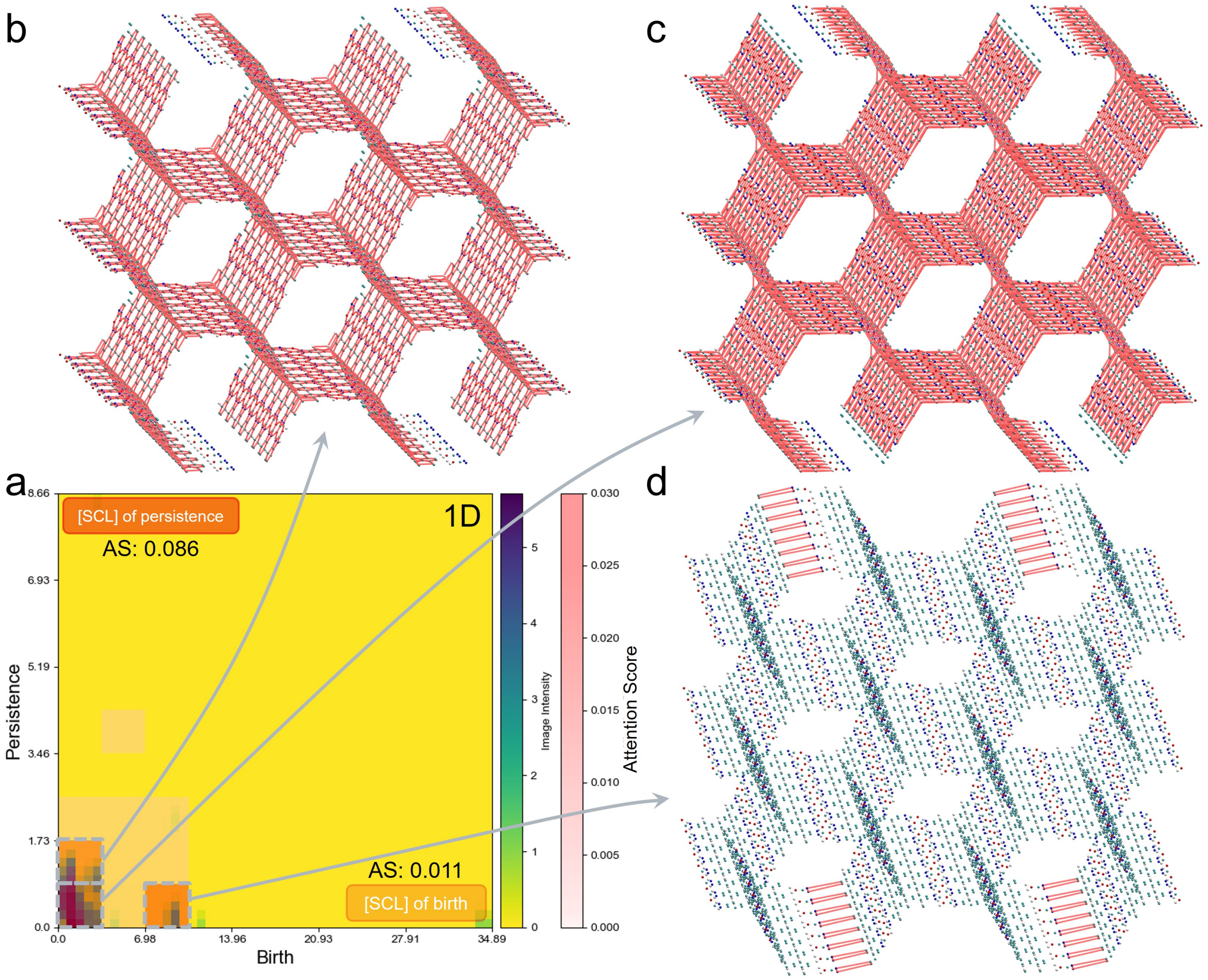

Fig. S12 Representative cycles of three patches with relatively high attention scores in the 1D persistence image of T2-γ. **a**, 1D persistence images of T2-γ with attention scores from fine-tuned MCRT that predicts CH_4 capacity. **b**, **c** and **d**, The representative cycles of topological objects within the patches with large attention scores.

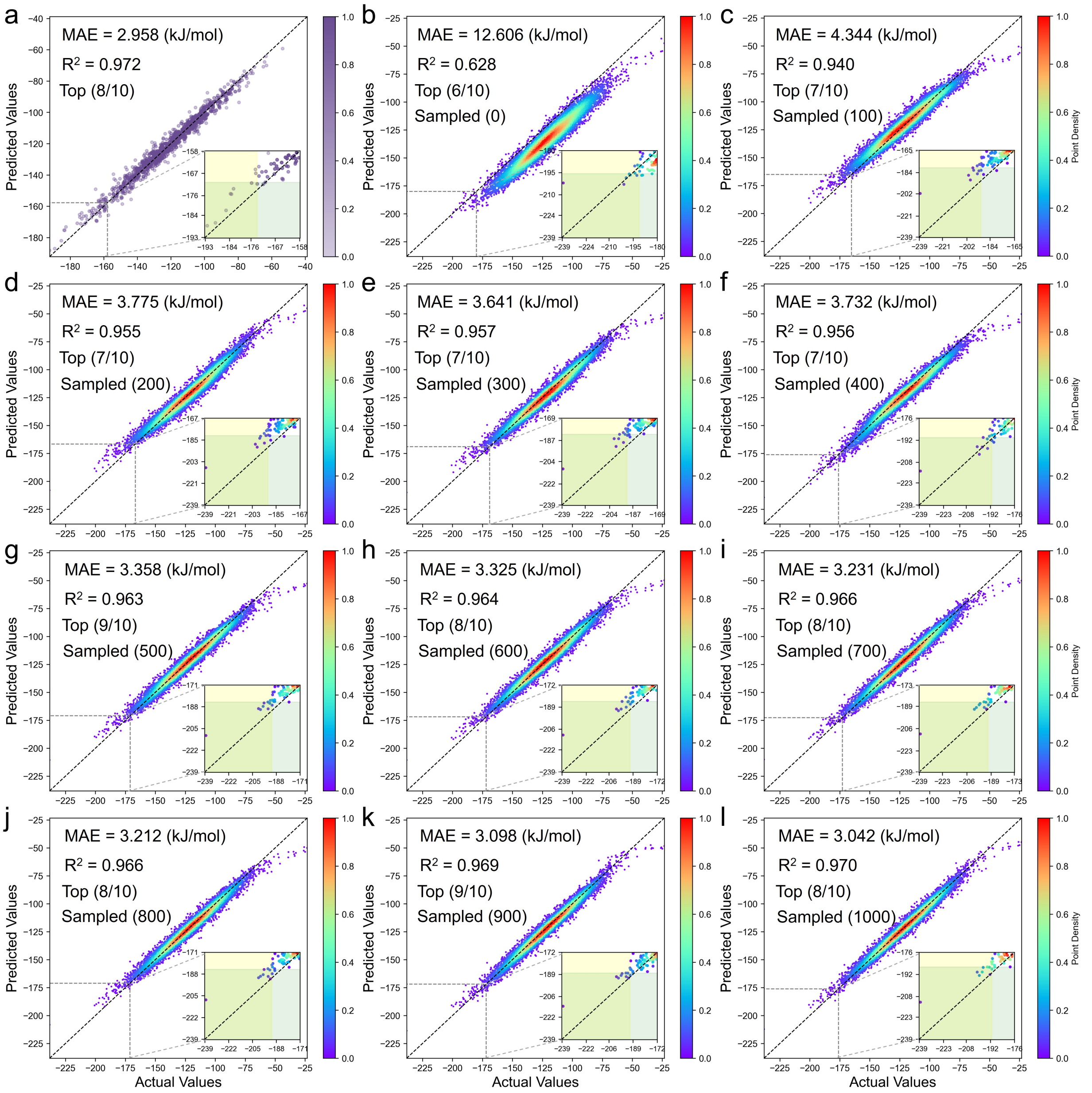

Fig. S13 Prediction results on test set of fine-tuned MCRT. **a**, LE_T2 (8k). **b-l**, The few-shot prediction results on test sets of fine-tuned MCRT for T2 with 0 (**b**), 100 (**c**), 200 (**d**), 300 (**e**), 400 (**f**), 500 (**g**), 600 (**h**), 700 (**i**), 800 (**j**), 900 (**k**), 1000 (**l**) samples.

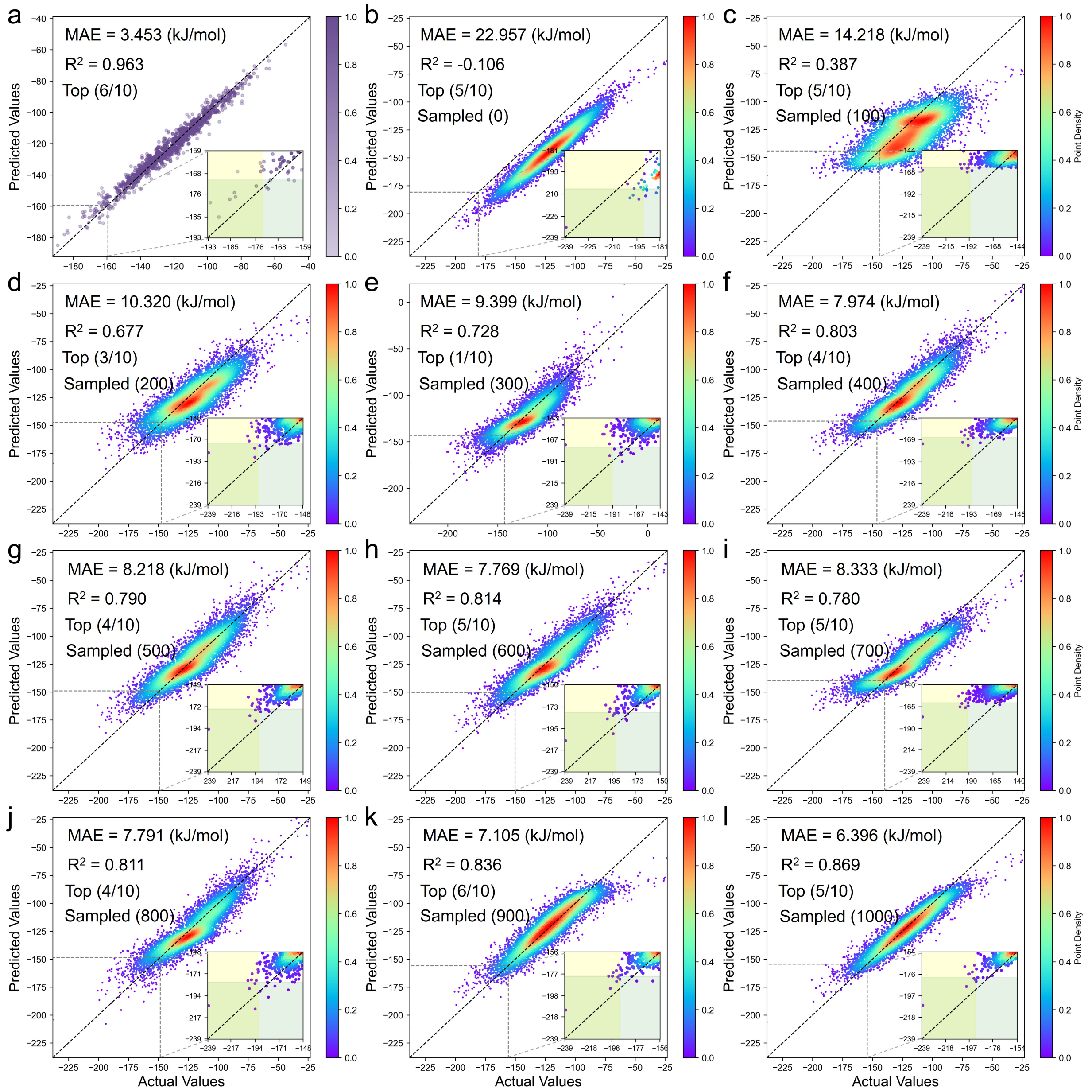

Fig. S14 Prediction results on test set of ALIGNN. **a**, LE_T2 (8k). **b-l**, The few-shot prediction results on test sets of fine-tuned ALIGNN for T2 with 0 (**b**), 100 (**c**), 200 (**d**), 300 (**e**), 400 (**f**), 500 (**g**), 600 (**h**), 700 (**i**), 800 (**j**), 900 (**k**), 1000 (**l**) samples.

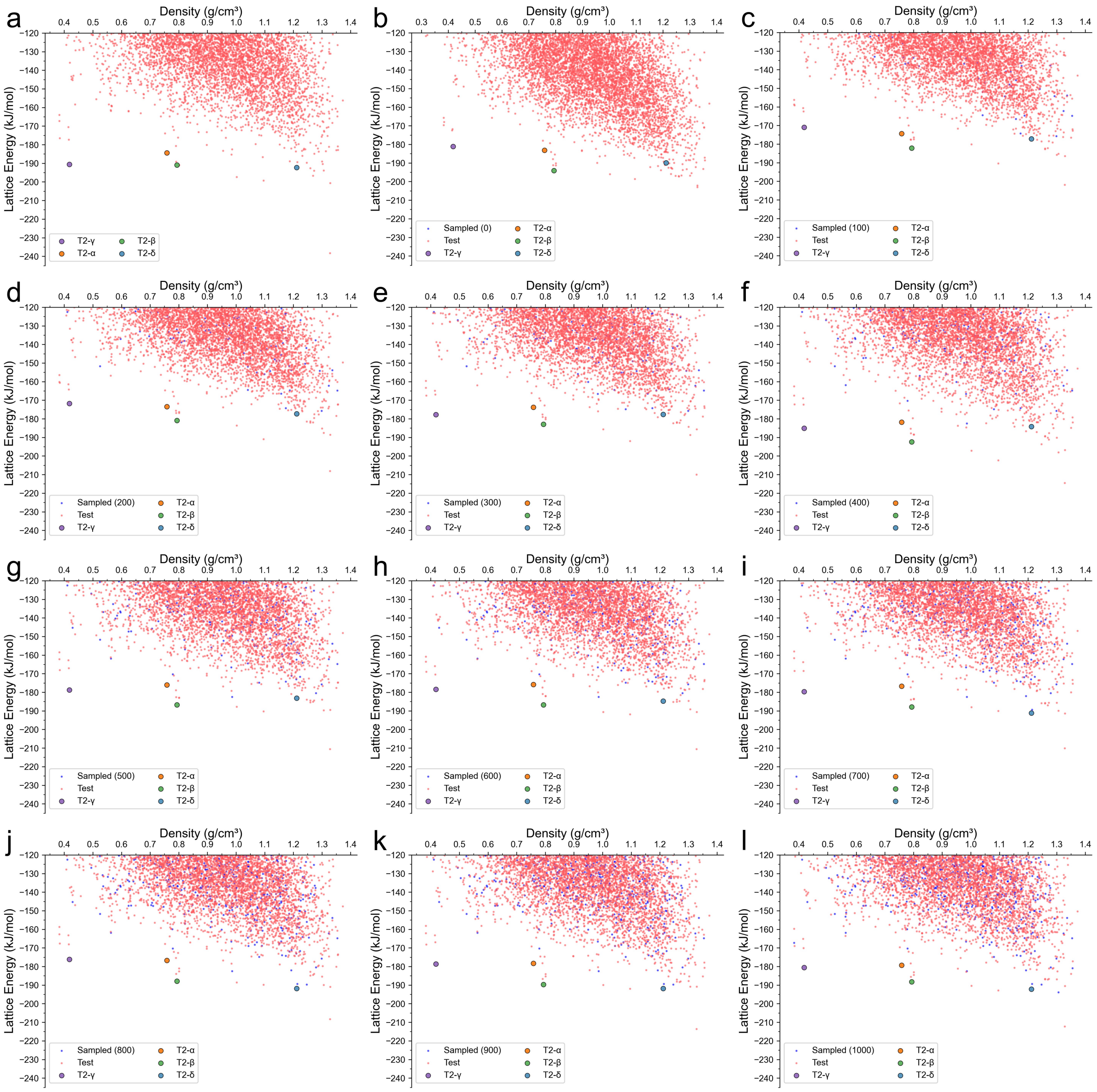

Fig. S15 MCRT-derived CSP energy–density plots. a, CSP energy–density plots for T2. **b-l**, Predicted CSP energy–density plots using MCRT for T2 with 0 (**b**), 100 (**c**), 200 (**d**), 300 (**e**), 400 (**f**), 500 (**g**), 600 (**h**), 700 (**i**), 800 (**j**), 900 (**k**), 1000 (**l**) samples. Only structures with a lattice energy of less than -120 kJ/mol are plotted. Even after zero-shot learning, **b**, the four key experimentally-observed porous structures for T2 are identified as being on the leading edge of the energy-density landscape, and 'spikes' emerge that correspond to families of related 1-D porous crystals, as observed in CSP plots generated with forcefield or DFT enery calculations (*Nature* **543**, 657 (2017); *Nature* **630**, 102 (2024)).

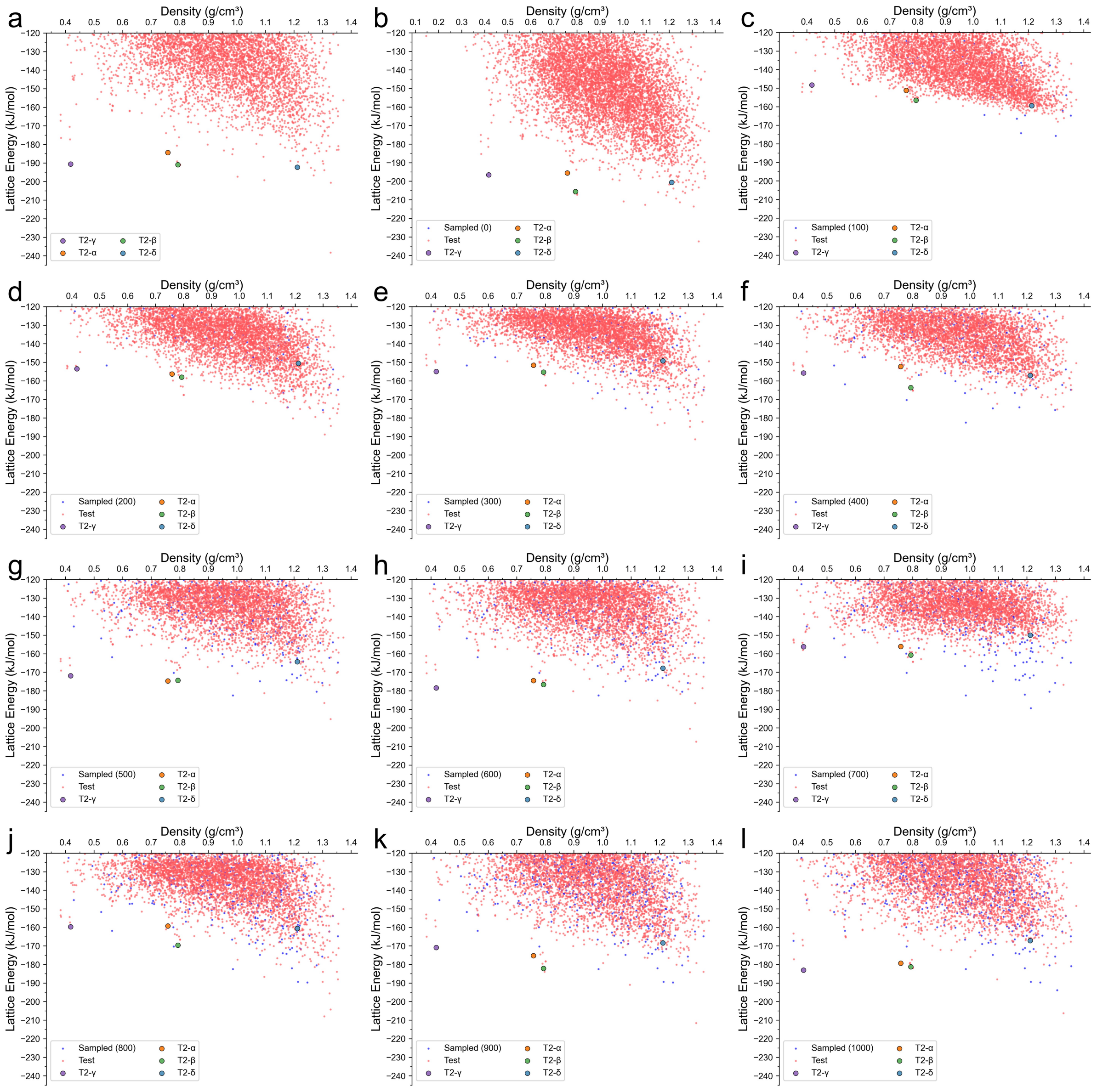

Fig. S16 ALIGNN-derived CSP energy–density plots. **a**, CSP energy–density plots for T2. **b-l**, Predicted CSP energy–density plots using ALIGNN for T2 with 0 (**b**), 100 (**c**), 200 (**d**), 300 (**e**), 400 (**f**), 500 (**g**), 600 (**h**), 700 (**i**), 800 (**j**), 900 (**k**), 1000 (**l**) samples. Only structures with a lattice energy of less than -120 kJ/mol are plotted.
